# MiR-146a-5p Mimic Inhibits NLRP3 Inflammasome Downstream Inflammatory Factors and CLIC4 in Neonatal Necrotizing Enterocolitis

**DOI:** 10.3389/fcell.2020.594143

**Published:** 2021-01-28

**Authors:** Jianglong Chen, Tong Chen, Jin Zhou, Xiuhao Zhao, Qingfeng Sheng, Zhibao Lv

**Affiliations:** Department of General Surgery, Shanghai Children's Hospital, Shanghai Jiao Tong University, Shanghai, China

**Keywords:** neonatal necrotizing enterocolitis, microRNA, NLRP3 inflammasomes, CLIC4, macrophage

## Abstract

**Objective:** Necrotizing enterocolitis (NEC) is a gastrointestinal emergency with a severe inflammation storm, intestinal necrosis, and perforation. MicroRNA-146a-5p (miR-146a-5p) has been reported to be a valuable anti-inflammatory factor in various intestinal inflammatory disorders. However, the role of miR-146a-5p in NEC, its effects on nucleotide-binding domain and leucine-rich repeat-containing protein 3 (NLRP3) inflammasome, and its downstream inflammatory factors remain unknown. This study aimed to investigate the role of miR-146a-5p and NLRP3 inflammasome and its downstream inflammatory factors in NEC development.

**Methods:** The expression levels of miR-146a and NLRP3 inflammasome were investigated in intestinal tissues. Next, the mechanism by which miR-146a-5p regulates NLRP3 inflammasome activation was explored *in vitro* in THP-1 cells. Finally, to identify the effects of miR-146a-5p on NEC *in vivo*, NEC mice were transinfected with miR-146a-5p overexpression adenovirus before the occurrence of NEC.

**Results:** NLRP3 inflammasome enzymatic protein caspase-1 and its downstream inflammatory factors increased in NEC intestinal samples in both humans and mice, and miR-146a-5p expression level was increased and mainly expressed in the macrophages of the affected intestine. *In vitro*, only miR-146a-5p mimic inhibited NLRP3 inflammasome downstream inflammatory factors and its upstream protein chloride intracellular channel protein 4 (CLIC4) expression in cellular membrane in the THP-1 cell line, and this only occurred under mild/moderate LPS concentration. MiR-146a-5p overexpression adenovirus transfection reduced CLIC4 cellular membrane expression and inhibited NLRP3 downstream factors increasing *in vivo*. After the transfection of miR-146a-5p adenovirus, the survival rate of NEC mice was increased, and intestinal injury was ameliorated.

**Conclusion:** MiR-146a-5p inhibited NLRP3 inflammasome downstream inflammatory factors and CLIC4 membrane expression in NEC. Additionally, miR-146a-5p could attenuate inflammation and intestinal injury in the NEC-affected intestine.

## Introduction

Necrotizing enterocolitis (NEC) is one of the most serious and common digestive system emergencies in premature infants and is characterized by an inflammation storm as well as intestinal necrosis and perforation. Previous studies have revealed that NEC is characterized by a macrophage-rich inflammatory infiltrate (Maheshwari et al., 2011; MohanKumar et al., 2012), and gut macrophage populations are normally maintained through continuous recruitment of circulating monocytes and *in situ* differentiation of these cells in the lamina propria (Smythies et al., 2006; Maheshwari et al., 2009). Macrophages are the first component of the innate immune response.

MicroRNAs (miRNAs) are non-coding endogenous small RNAs containing 19–25 nucleotides that regulate post-transcriptional silencing of target genes. MicroRNA-146a (miR-146a) is a valuable negative regulator of innate immune response that functions by controlling the lipopolysaccharide (LPS) Toll-like 4 receptor signaling pathway and targeting TRAF6 and IRAK1 (Tang et al., 2009; Chassin et al., 2010, 2012; Saba et al., 2014; Chen et al., 2018). Furthermore, miR-146a plays a vital role in the macrophage-induced innate immune response (Jiang et al., 2012), and its dysregulation has been observed in autoimmune diseases (Nakasa et al., 2008; Tang et al., 2009). Accumulating evidence shows that miR-146a is involved in the intestinal innate immune response, particularly in neonates (Chassin et al., 2010; Wu et al., 2019). However, the role of miR-146a in NEC innate immunity is still unclear.

Pattern recognition receptors (PPRs) include two major classes according to their sublocation in the cell. Consisting of Toll-like receptors (TLRs) and C-type lectin receptors (CLRs), PPRs are found in the plasma membrane and endosomes, where they can survey PAMPs (pathogen-associated molecular patterns) and DAMPs (danger-associated molecular patterns) in the extracellular milieu. A second class of PRRs resides in intracellular compartments and includes the RIG-I-like receptor (RLR), the AIM2-like receptor (ALR), and the nucleotide-binding domain and leucine-rich repeat-containing (NLR) proteins (Lamkanfi and Dixit, 2014). Nucleotide-binding domain and leucine-rich repeat-containing protein 3 (NLRP3), an NLR, is activated by the production of mitochondrial reactive oxygen species (Zhou et al., 2011), the release of mitochondrial DNA (Shimada et al., 2012), the cytosolic release of lysosomal cathepsins, and K^+^ efflux (Munoz-Planillo et al., 2013). Recent studies show that NLRP3 inflammasome play a role in NEC development (Fan et al., 2019; Yu et al., 2019), but the mechanism is still unknown.

Chloride intracellular channel (CLIC) proteins participate in a number of biological functions, including membrane potential regulation, cell volume regulation, cell proliferation, and apoptosis (Jentsch and Pusch, 2018). CLIC4 is a member of the chloride intracellular channel protein family, in which structure is evolutionarily conserved and belongs to the glutathione S-transferase (GST)-fold superfamily. CLIC4 knockdown was found to impair the NLRP3 inflammasome activation in bone marrow-derived macrophages under LPS/ATP stimulation (Domingo-Fernandez et al., 2017). In addition, NLRP3 inflammasome activation can be blocked by several non-specific chloride channel inhibitors, ATP-induced caspase-1 activation, and IL-1β production, which is enhanced in macrophages after chloride-free medium incubation (Verhoef et al., 2005; Compan et al., 2012; Daniels et al., 2016).

Collectively, it can be hypothesized that miR-146a-5p may play a role in NEC development, and NLRP3 inflammasome could be closely correlated with CLIC4. In this study, we investigated the expression levels of miR-146a and NLRP3 inflammasome and its downstream inflammatory factors. The mechanism by which miR-146a-5p regulates the activation of NLRP3 inflammasome was also explored. Furthermore, miR-146a-5p's *in vivo* effects in NEC were evaluated.

## Materials and Methods

### Human Samples

Intestinal samples from 17 NEC children (average age: 7.04 days, median age: 3 days) and 22 children with intestinal atresia (IA) (average age: 13.47 days, median age: 7 days) were collected in the General Surgery Department of Shanghai Children's Hospital from June of 2014 to December of 2018. Among these NEC children's intestinal samples, both necrotic and uninflamed intestinal tissues were collected. For IA samples, only uninflamed intestinal tissues were collected. The acquisition of the tissue samples was approved by the Institutional Review Board at Shanghai Children's Hospital, and written informed consent was obtained from each patient's parents before inclusion in the study.

### Mice and Treatment

C57BL/6J mice (7 days old) weighing about 4 g were purchased from Shanghai Jiesijie Experimental Animal Co., Ltd. (Shanghai, China). NEC was induced using formula gavage 6 times/day (15 g Similac Advance infant formula [Abbott Laboratories, Chicago, USA] in 75 ml canine milk replacement, using a 1.9-French angio-catheter placed into the mouse esophagus under direct vision), as well as hypoxia (99% N2) for 60 s and cold (4°C) for 10 min in a hypoxic chamber (Billups-Rothenberg, CA, USA) twice daily for 4 days. The protocol was partly modified as described previously (Leaphart et al., 2007a; Sodhi et al., 2010; Afrazi et al., 2014). Several studies have shown that a NEC animal model can be induced by this protocol that resembles human NEC (Nadler et al., 2000; Cetin et al., 2004; Qureshi et al., 2005; Leaphart et al., 2007b). Control mice were fed by a dam. Terminal ileum samples were harvested as soon as animals died from NEC or were euthanized by decapitation at the end of the experimental period for subsequent analysis. All experiments were approved by the Institutional Review Board at our hospital.

### MiR-146a-5p Adenovirus Transduction *In vivo*

Adenovirus was constructed by Shanghai Obio Technology company (Shanghai, China). Positive adenovirus was pDKD-CMV-mcherry-U6-miR30(mmu-miR-146a-5p), and scrambled adenovirus was pDKD-CMV-mcherry-U6-shRNA. NEC and control mice were divided into four groups for adenovirus intraperitoneal injection: control mice injected with scrambled adenovirus (Scrambled +ctrl), NEC mice injected with scrambled adenovirus (Scrambled +NEC), control mice injected with positive adenovirus (miR-146a-5p-OE+ctrl), and NEC mice injected with positive adenovirus (miR-146a-5p-OE+NEC). A total volume of 50 μl containing 1.58 × 10^9^ TU adenoviruses was intraperitoneally injected 24 h before NEC was conducted, which is in accordance with previous research (Nomura et al., 2006). Animals' daily body weight and survival times were recorded. Samples were harvested when animals died from NEC or were euthanized by decapitation at the end of the experimental period for subsequent analysis.

### Cell Culture and Treatment

THP-1 cells were kindly provided by Stem Cell Bank, Chinese Academy of Sciences. THP-1 cells were cultured in RPMI supplemented with 10% FBS (fetal bovine serum) and 50 μM 2-mercaptoethanol (Sigma-Aldrich, MO, USA), in a humidified atmosphere of 37°C and 5% CO_2_. For experiments, a total of 1 × 10^6^ cells were plated on a 12-well plate overnight. THP-1 cells were differentiated at 24 h using 0.5 mM phorbol 12-myristate 13-acetate (PMA) (Sigma-Aldrich, MO, USA). The following day, medium was replaced by Opti-DMEM, and cells were pre-incubated with 80 nM miR-146a-5p overexpression sequence (mimic: sense: 5′-UGAGAACUGAAUUCCAUGGGUU-3′, antisense: 5′-CCCAUGGAAUUCAGUUCUCAUU-3′) or miR-146a-5p knockdown sequence (inhibitor: 5′-AACCCAUGGAAUUCAGUUCUCA-3′) (Gene-Pharma, Shanghai, China), respectively, and their scrambled control sequences were preincubated under the same conditions (mimic scrambled: sense: 5′-UUCUCCGAACGUGUCACGUTT-3′, antisense: 5′-ACGUGACACGUUCGGAGAATT-3′), inhibitor scrambled sequence: (nc-in: 5′-CAGUACUUUUGUGUAGUACAA-3′) (Gene-Pharma, Shanghai, China). After 48 h, cells were treated for 3 h with 0.5 μg/ml LPS (Sigma-Aldrich, MO, USA) and 5 mM ATP (Sigma-Aldrich, MO, USA), 1.0 μg/ml LPS and 5 mM ATP, and 10 μg/ml LPS and 5 mM ATP, respectively, and ATP was added during the last 30 min of LPS stimulation. The supernatant medium and treated cells were collected for subsequent analysis.

### Histopathological Examination of Intestinal Samples

The collected terminal ileum samples were fixed in 4% paraformaldehyde solution, embedded in paraffin, cut into sections, stained with hematoxylin and eosin (HE), and pathologically graded according to the published scoring criteria by two expert pathologists who were blind to the experimental groups (Sheng et al., 2013, 2014; Ginzel et al., 2016). The grading system was as follows: Grade 0: normal ileum, Grade 1: mild injury to the tip of the villus, Grade 2: partial loss of the villus, Grade 3: severe submucosal injury, and Grade 4: complete necrosis. A grade equal to or >2 suggests the occurrence of NEC.

### MicroRNA Fluorescence *In situ* Hybridization (FISH) and Immunofluorescence Co-staining

Human samples and mouse terminal ileums were fixed in hybridization *in situ* fixing solution (Servicebio, Wuhan, China), embedded in paraffin, and cut into 5-μm-thick sections. Next, the sections underwent hybridization *in situ* with an RNA FISH kit (Gene-Pharma, Shanghai, China). Sections were deparaffinized twice and hydrated through decreasing concentrations of ethanol (100, 95, 90, 80, and 70%). After that, sections were incubated with proteinase K at 37°C for 20 min and hydrated again through increasing concentrations of ethanol (70, 80, 90, and 100%). Next, sections were denaturation at 78°C for 8 min and then were incubated with miR-146a-5p probe (sequences: 5′-AACCCATGGAATTCAGTTCTCA-3′-FAM) (Gene-Pharma, Shanghai, China) at 4°C overnight. Subsequently, sections were incubated with immunofluorescence primary antibody (F4/80: Abcam, MA, USA) at 4°C overnight and then with secondary antibody at room temperature for 2 h. Next, sections were stained with 4′,6-diamidino-2-phenylindole (DAPI). Finally, sections were observed under a fluorescence microscope by two expert pathologists who were blind to the experimental groups, and miR-146a-5p positive cells, miR-146a-5p, and F4/80 double-positive cells were counted in five random scope fields, and an average of five fields was calculated.

### Immunohistochemistry

For immunohistochemical staining, 5-μm-thick paraffin-embedded sections were deparaffinized and hydrated through decreasing concentrations of ethanol (100, 85, and 75%) after which peroxidase activity was blocked using 3% H_2_O_2_ in methanol for 15 min at room temperature. Heat-induced antigen retrieval was conducted in 10 mM sodium citrate solution (pH = 6.0) for about 2 min until boiling in a microwave. Sections were washed three times with PBS and blocked with 5% bovine serum albumin for 60 min at room temperature. Sections were incubated overnight at 4°C with primary antibodies specific for NLRP3 and Caspase-1 (Abcam, Cambridge, United Kingdom), followed by incubation with horseradish peroxidase (HRP)-coupled secondary antibodies with 50 mM Tris-HCl buffer (pH 7.4) at room temperature for 1 h. The color was developed by a 15 min incubation at room temperature with DAB solution (Sangon Biotech Co., Ltd., Shanghai, China), and then the sections were weakly counterstained with hematoxylin for 10 min at room temperature. Negative controls were included using the replacement of the primary antibody with PBS. The sections were lightly counterstained with hematoxylin.

### Enzyme-Linked Immunosorbent Assay (ELISA) Analysis

The supernatant medium from the treated THP-1 cells was collected, and IL-1β, IL-6, IL-10, IL-18, and TNF-α were measured using the appropriate ELISA kits (Lianke Bio Co., Ltd., Hangzhou, China).

### Western Blotting

Total protein was isolated from cell and tissues using a radioimmunoprecipitation assay (RIPA) lysis buffer (Thermo Scientific, Waltham, MA, USA). Subcellular fraction protein was extracted using a membrane, nuclear, and cytoplasmic protein extraction kit (Sangon Biotech Co., Ltd., Shanghai, China). Protein concentrations of samples were determined using the BCA Protein Assay Kit (Thermo Scientific). Then, protein samples were subjected to SDS-PAGE and transferred to polyvinylidene difluoride (PVDF) membranes, which were incubated in 5% BSA for 1 h at room temperature. Next, PVDF membranes were incubated overnight at 4°C with the following primary antibodies: NLRP3 (Abcam, MA, USA), pro-Caspase-1 (Santa Cruz Biotechnology, TX, USA), Caspase-1 p10 (Santa Cruz Biotechnology, TX, USA), β-actin (Abcam), CLIC4 (Abcam), histone H3 (Proteintech, Rosemont, USA), and N-cadherin (Abcam). Next, membranes were incubated with IgG HRP-conjugated secondary antibodies (Cell Signaling Technology, MA, USA) for 2 h at room temperature. Protein bands were detected using a ChemiDoc-It system (Tanon, Shanghai, China) and an ECL kit (Bio-Rad, CA, USA). Protein levels were determined using ImageJ (National Institutes of Health, MD, USA). The density of each band was normalized to its respective loading control (β-actin, histone H3, and N-cadherin).

### RNA Extraction and Quantitative Real-Time PCR

Total RNA was extracted from the treated THP-1 cells using Trizol (Life Technologies, CA, USA) following the manufacturer's instructions. For miR-146a-5p, first-strand cDNA was synthesized using a stem-loop method reverse transcription kit (Sangon Biotech Co., Ltd.), and other genes' cDNA were reversed using a Takara reverse transcription kit (Takara Bio, CA, USA). A quantitative mRNA kit (Takara Bio) and microRNA qPCR kit (Sangon Biotech Co., Ltd.) were used for mRNA and microRNA quantitative real-time PCR analysis, respectively, using the SYBR Green fluorescence system (Roche, MA, USA). The following primers were used:

β-actin: forward: 5′-TCGTGCGTGACATTAAGGAGAAGC-3′,

reverse: 5′-GGCGTACAGGTCTTTGCGGATG-3′

U6: forward: 5′-AGAGAAGATTAGCATGGCCCCTG-3′,

reverse: 5′-ATCCAGTGCAGGGTCCGAGG-3′

NLRP3: forward: 5′-ATGCTGCCTGTTCTCATGGATTGG-3′,

reverse: 5′-GCTTCTGGTTGCTGCTGAGGAC-3′

Caspase-1: forward: 5′-ATGGACAAGTCAAGCCGCACAC-3′

reverse: 5′-TCCCACAAATGCCTTCCCGAATAC-3′

CLIC4: forward: 5′-GGCCAGAGGCTAATGAAGCACTG-3′,

reverse: 5′-GGCCACCACCTTGACAATATGCAG-3′

IL-1β: forward: 5′-GCGGCATCCAGCTACGAATCTC-3′,

reverse: 5′-CGGAGCGTGCAGTTCAGTGATC-3′

IL-18: forward: 5′-TGGCTGCTGAACCAGTAGAAGA-3′,

reverse: 5′-TGGTCCGGGGTGCATTATCT-3′

IL-6: forward: 5′-GGTGTTGCCTGCTGCCTTCC-3′,

reverse: 5′-GCTCTGGCTTGTTCCTCACTACTC-3′

IL-10: forward: 5′-ACTGCTCTGTTGCCTGGTCCTC-3′,

reverse: 5′-GCCTTGATGTCTGGGTCTTGGTTC-3′

TNF-α: forward: 5′-TGCTCCTCACCCACACCATCAG-3′,

reverse: 5′-TCCCAAAGTAGACCTGCCCAGAC-3′

miR-146a-5p: forward: 5′-CGCGTGAGAACTGAATTCCA-3′,

reverse: 5′-AGTGCAGGGTCCGAGGTATT-3′

Specific mRNA expression or miR-146a-5p levels were normalized relative to β-actin mRNA or U6 levels, respectively, using the comparative 2^−Δ*ΔCt*^ method.

### Statistical Analysis

Statistical analysis was performed using the SPSS21.0 statistical software. The descriptive data are expressed as mean ± SD. The groups were compared using a *t-*test when two groups were compared, or a one/two-way analysis of variance (ANOVA) when more than two groups were compared. The log-rank test was used for survival analysis. Differences at a value of *P* < 0.05 were considered statistically significant.

## Results

### MiR-146a-5p Expression Level Is Increased in NEC Human and Mouse Intestinal Tissues

**Figure 1:**
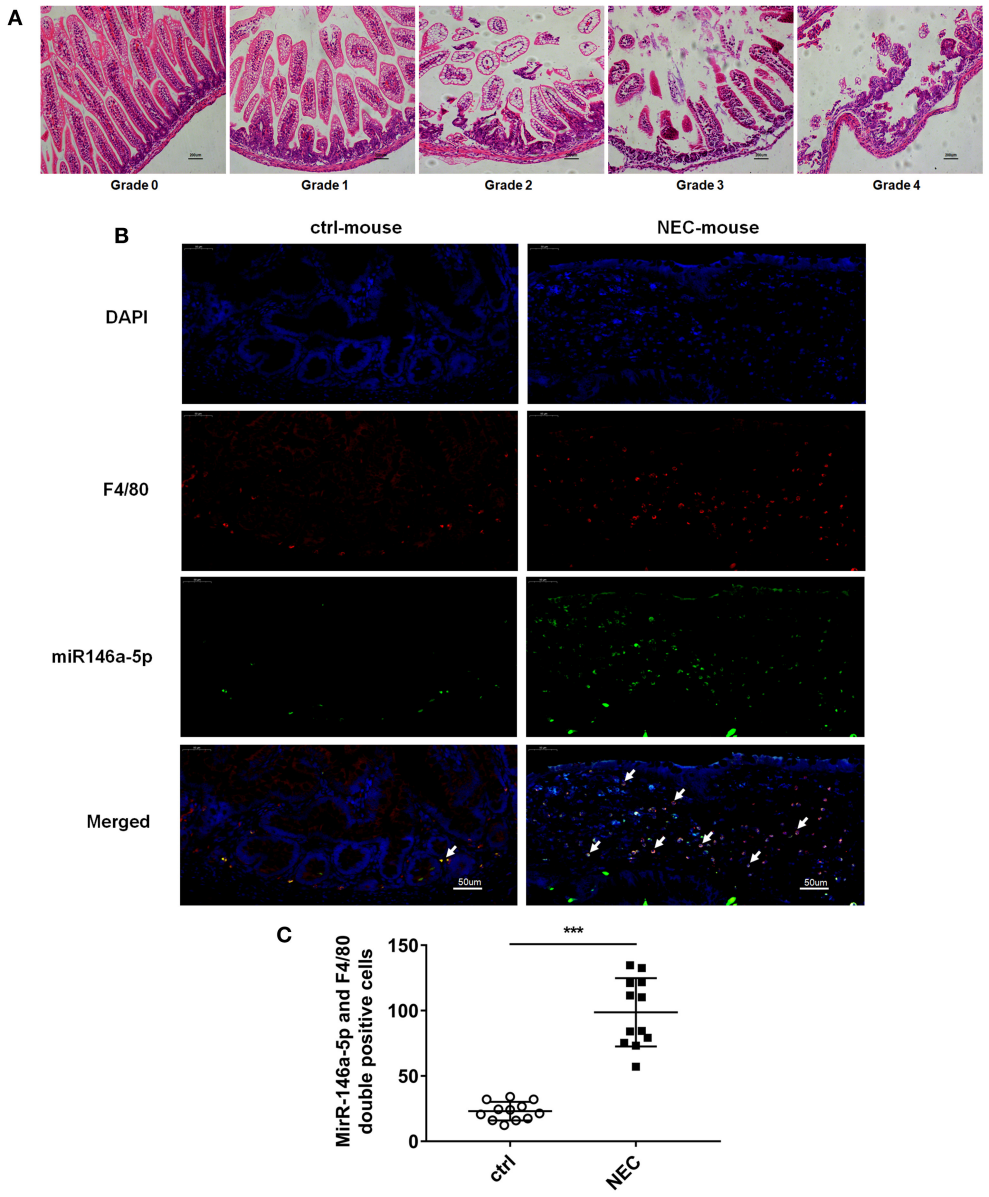
MiR-146a-5p and F4/80 double-positive cells in the intestines of NEC mice. **(A)** Pathological grade of terminal ileum according to HE, Grade 0: normal ileum; Grade 1: mild injury to the tip of the villus; Grade 2: partial loss of the villus; Grade 3: severe submucosal injury; and Grade 4: complete necrosis. **(B)** FISH and immunofluorescence merged representative pictures of miR-146a-5p and F4/80 double-positive cells in intestinal tissues extracted from control and NEC mice. **(C)** The comparison of miR-146a-5p and F4/80 double-positive cell numbers in intestinal tissues between control and NEC mice (*n* = 12 per group). ***P < 0.001.

**Figure 2:**
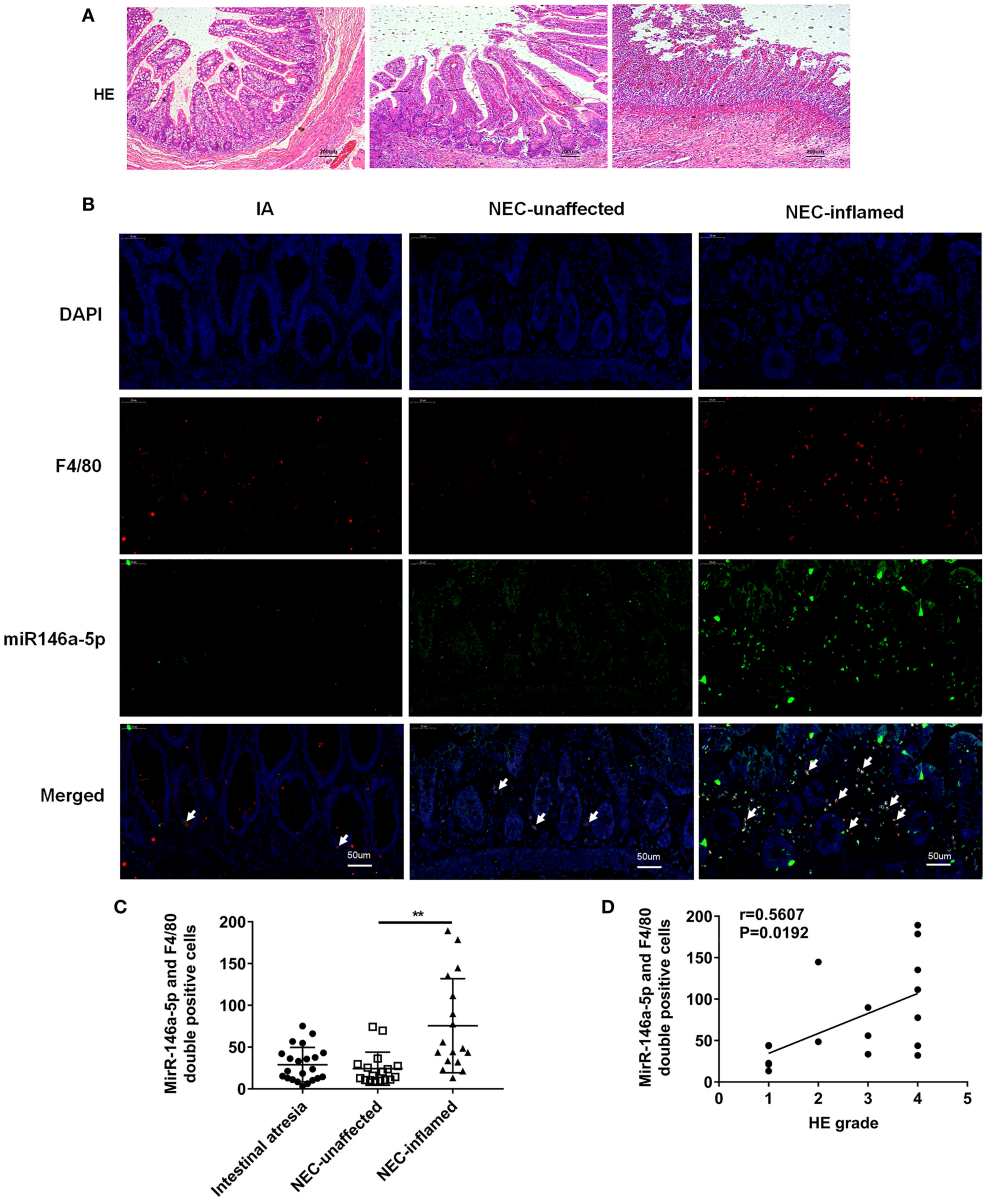
MiR-146a-5p and F4/80 double-positive cells in the intestines of children with intestinal atresia and NEC. **(A)** HE representative pictures of intestinal tissue from children with intestinal atresia and NEC. **(B)** FISH and immunofluorescence merged representative pictures of miR-146a-5p and F4/80 double-positive cells in intestinal tissues of children with intestinal atresia and NEC. **(C)** The comparison of miR-146a-5p and F4/80 double-positive cell numbers in intestinal tissue between children with intestinal atresia (*n* = 22) and NEC (*n* = 17). **(D)** The correlation of miR-146a-5p and F4/80 double-positive cell number with HE grade in intestinal samples of children with NEC (*n* = 17). IA, intestinal atresia; NEC-unaffected, NEC patients' unaffected intestinal samples; NEC-inflamed, NEC patients' inflamed intestinal samples. **P < 0.01.

The pathological grade scores of NEC mouse terminal ileum intestine were assessed and ranged according to HE as follows: Grade 0: normal ileum, Grade 1: mild injury to the tip of the villus, Grade 2: partial loss of the villus, Grade 3: severe submucosal injury, and Grade 4: complete necrosis. A grade equal to or higher than 2 suggested the occurrence of NEC (Figure 1A). In previous studies, miR-146a-5p played a key role in regulating macrophage function. The miR-146a-5p was detected by FISH in the intestinal tissues of NEC mice. The miR-146a-5p was merged with F4/80 detecting by immunofluorescence, and the miR-146a-5p and F4/80 double-positive cells were counted. We found that miR-146a-5p was significantly increased in the NEC group (Figures 1B,C). The degree of intestinal destruction varied greatly between inflammatory and unaffected intestine. We assessed the pathological grade score of NEC inflammatory and unaffected intestine by HE (Figure 2A). The level of miR-146a-5p and F4/80 double-positive cells was significantly higher in the NEC inflamed intestine than in the unaffected intestine and samples showing intestinal atresia (Figures 2B,C). The number of miR-146a-5p and F4/80 double-positive cells also demonstrated positive correlation to the HE pathological grade score in the NEC human samples (Figure 2D).

### NLRP3 Inflammasome Downstream Inflammatory Factors Increased in NEC Human and Mouse Intestinal Tissues

**Figure 3:**
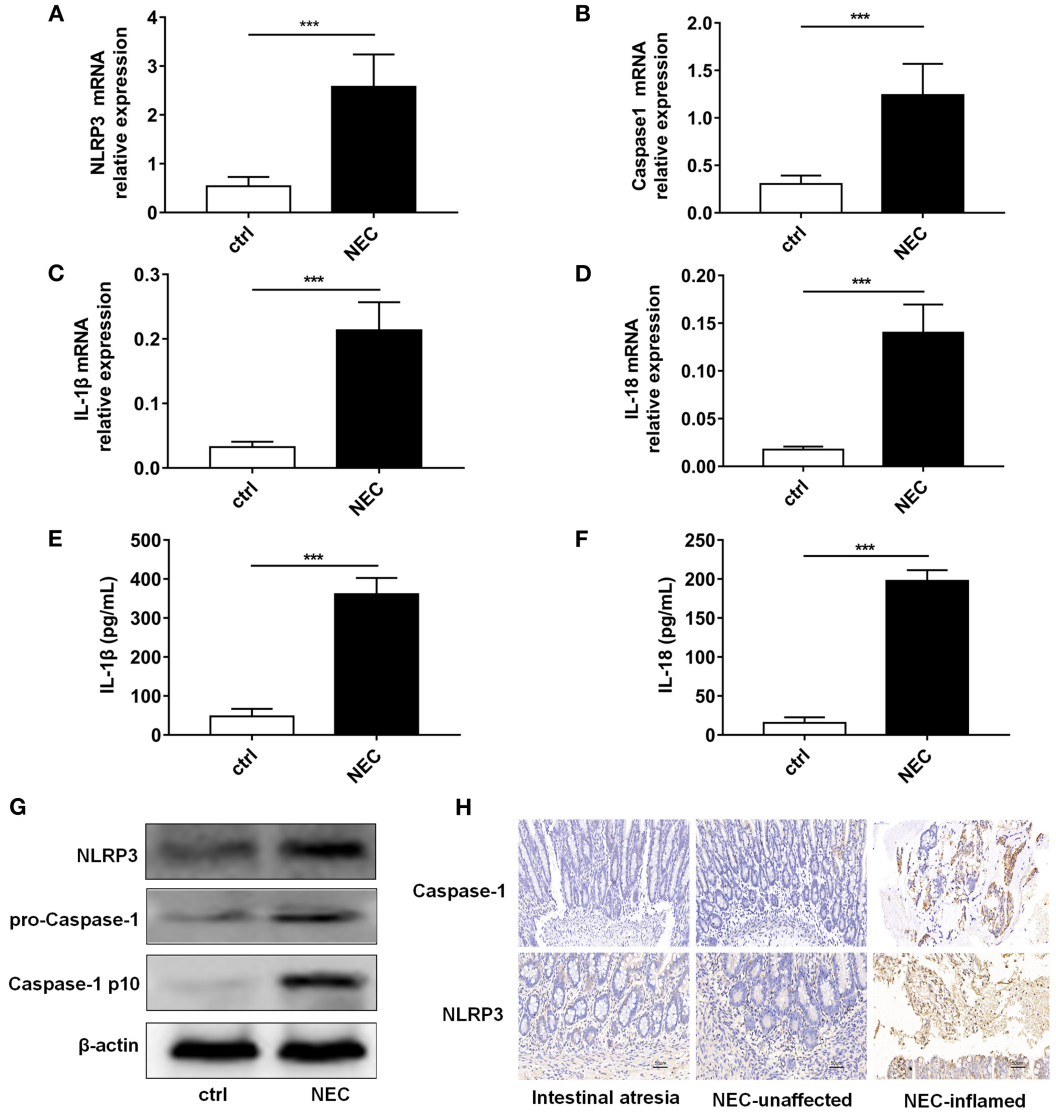
NLRP3 inflammasome activation level in NEC intestinal tissues. The mRNA expression levels of **(A)** NLRP3, **(B)** Caspase-1, **(C)** IL-1β, and **(D)** IL-18 level in intestinal tissue of control and NEC mice (*n* = 5 per group). ELISA showed the **(E)** IL-1β and **(F)** IL-18 expression levels in intestinal tissue of control and NEC mice (*n* = 5 per group). **(G)** NLRP3, pro-Caspase-1, and Caspase-1 p10 protein expression levels in intestinal tissue of control and NEC mice. β-actin was re-used as the control image. **(H)** Immunohistochemistry of NLRP3 and Caspase-1 in intestinal tissue of children with intestinal atresia and NEC. Ctrl, control mice; NEC, NEC mice; NEC-unaffected, NEC patients' unaffected intestinal samples; NEC-inflamed, NEC children patients' inflammatory intestinal samples. ***P < 0.001.

NLRP3 inflammasome activation was reported to be an important regulator in NEC (Yin et al., 2020). Thus, we detected the expression level of NLRP3 inflammasome relative proteins, and their downstream inflammatory factors IL-1β and IL-18. Our results showed that mRNA of NLRP3, Caspase-1, IL-1β, and IL-18 was higher in the NEC group than that in the control group (Figures 3A–D). ELISA showed that IL-1β and IL-18 both increased in NEC (Figures 3E,F). The protein expression levels of NLRP3, pro-Caspase-1, and Caspase-1 p10 were all higher in the NEC mice (Figure 3G). In children's samples, both NLRP3 and Caspase-1 were increased in NEC, and they were expressed both in the epithelium and lamina propria (Figure 3H).

The above data demonstrated that NLRP3 inflammasome enzymatic protein caspase-1, as well as their downstream IL-1β and IL-18, were increased in NEC.

### MiR-146a-5p Overexpression Decreased LPS/ATP-Induced NLRP3 Inflammasome Downstream Inflammatory Factors in Macrophages Only If LPS Concentration Was Relatively Low

**Figure 4:**
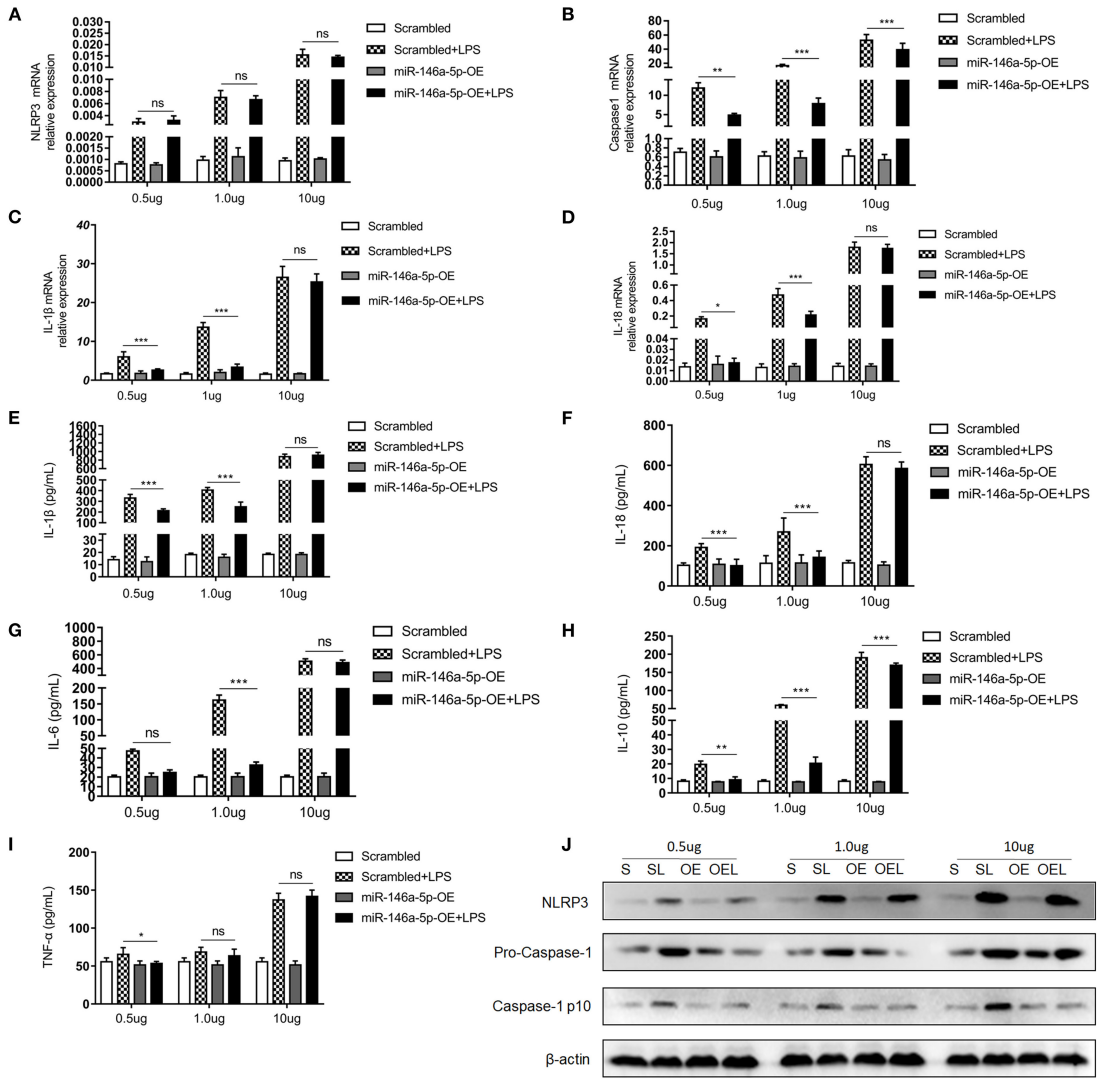
The effect of miR-146a-5p overexpression on the NLRP3 inflammasome activation level in macrophage after LPS/ATP stimulation. **(A–D)** mRNA expression levels of NLRP3, Caspase-1, IL-1β, and IL-18 after mimic preincubation followed by 0.5 μg/ml LPS + 5 mM ATP or 1.0 μg/ml LPS + 5 mM ATP or 10 μg/ml LPS + 5 mM ATP stimulation (*n* = 6 per group). **(E–I)** ELISA showing IL-1β, IL-18, IL6, IL-10, and TNF-a expression levels after mimic pre-incubation followed by 0.5 μg/ml LPS + 5 mM ATP or 1.0 μg/ml LPS + 5 mM ATP or 10 μg/ml LPS + 5 mM ATP stimulation (*n* = 6 per group). **(J)** NLRP3, pro-Caspase-1, and Caspase-1 p10 protein expression levels after mimic pre-incubation followed by 0.5 μg/ml LPS + 5 mM ATP, 1.0 μg/ml LPS + 5 mM ATP, or 10 μg/ml LPS + 5 mM ATP stimulation. β-actin was re-used as the control image. Scrambled (S), miR-146a-5p scrambled sequence; Scrambled +LPS (SL), miR-146a-5p scrambled sequence +LPS; miR-146A-5p-OE (OE), miR-146A-5p overexpression mimic; miR-146A-5p-OE+LPS (OEL), miR-146A-5p overexpression mimic +LPS. *P < 0.05, **P < 0.01, ***P < 0.001. ns, no statistical difference.

MiR-146a-5p was considered to be a negative regulator in innate immune responses in previous studies. In this study, Figure 2 show that miR-146a-5p-positive macrophages were associated with a worse pathological grade. Therefore, we investigated the role of miR-146a-5p mimics in the LPS/ATP-induced NLRP3 inflammasome activation. A THP-1 cell line was trans-ducted with miR-146a-5p overexpression sequence for 48 h before LPS/ATP stimulation. After miR-146a-5p overexpression, miR-146a-5p expression level was confirmed (Supplementary Figure 1). The results showed that NLRP3 inflammasome relative proteins, IL-1β, IL-18, IL-6, IL-10, and TNF-α were increased after LPS/ATP incubation (Figure 4). MiR-146a-5p overexpression had no significant effect on NLRP3 mRNA and protein expression (Figures 4A,J), but inhibited the mRNA expression of Caspase-1, IL-1β, and IL-18 at 0.5 and 1.0 μg of LPS, but not at 10 μg of LPS (Figures 4B–D). ELISA showed that IL-1β and IL-18 were downregulated by miR-146a-5p overexpression at 0.5 and 1.0 μg of LPS, but not at 10 μg of LPS (Figures 4E,F), while IL-6, IL-10, and TNF-α were downregulated by miR-146a-5p overexpression at a different degree at a gradient of LPS concentration (Figures 4G–I). In addition, protein levels of pro-Caspase-1 and Caspase-1 p10 were downregulated by miR-146a-5p overexpression, and NLRP3 showed no significant changes (Figure 4J).

Next, we determined the effect of miR-146a-5p knockdown in the LPS/ATP-induced NLRP3 inflammasome activation. However, miR-146a-5p knockdown showed no effects on the NLRP3 inflammasome or IL-1β and IL-18 mRNA (Supplementary Figures 2A–D) and protein (Supplementary Figure 2J) expression. Additionally, ELISA demonstrated that miR-146a-5p knockdown had no significant effect on NLRP3 downstream inflammatory factors IL-1β and IL-18, however, it promoted IL-10 and TNF-α expression (Supplementary Figures 2E–I).

All these data indicated that only miR-146a-5p overexpression decreased LPS/ATP-induced NLRP3 inflammasome enzymatic protein caspase-1 and downstream inflammatory factors expression.

### MiR-146a-5p Overexpression Decreased NLRP3 Inflammasome Upstream Protein CLIC4 Cellular Membrane Expression

**Figure 5:**
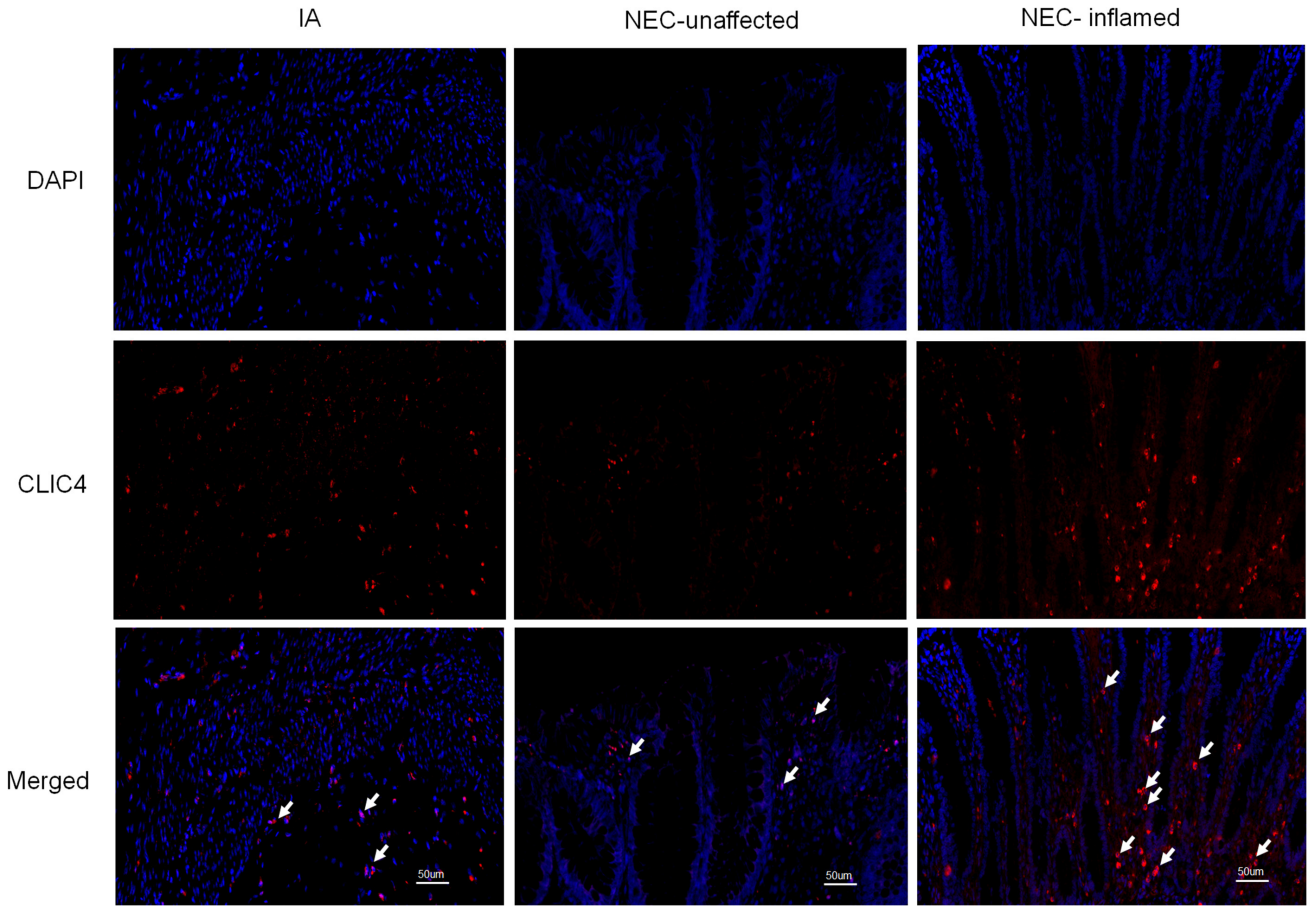
CLIC4 expression level in the intestines of children with intestinal atresia and NEC. IA, intestinal atresia; NEC-unaffected, NEC patients' unaffected intestinal samples; NEC-inflamed, NEC patients' inflamed intestinal samples.

**Figure 6:**
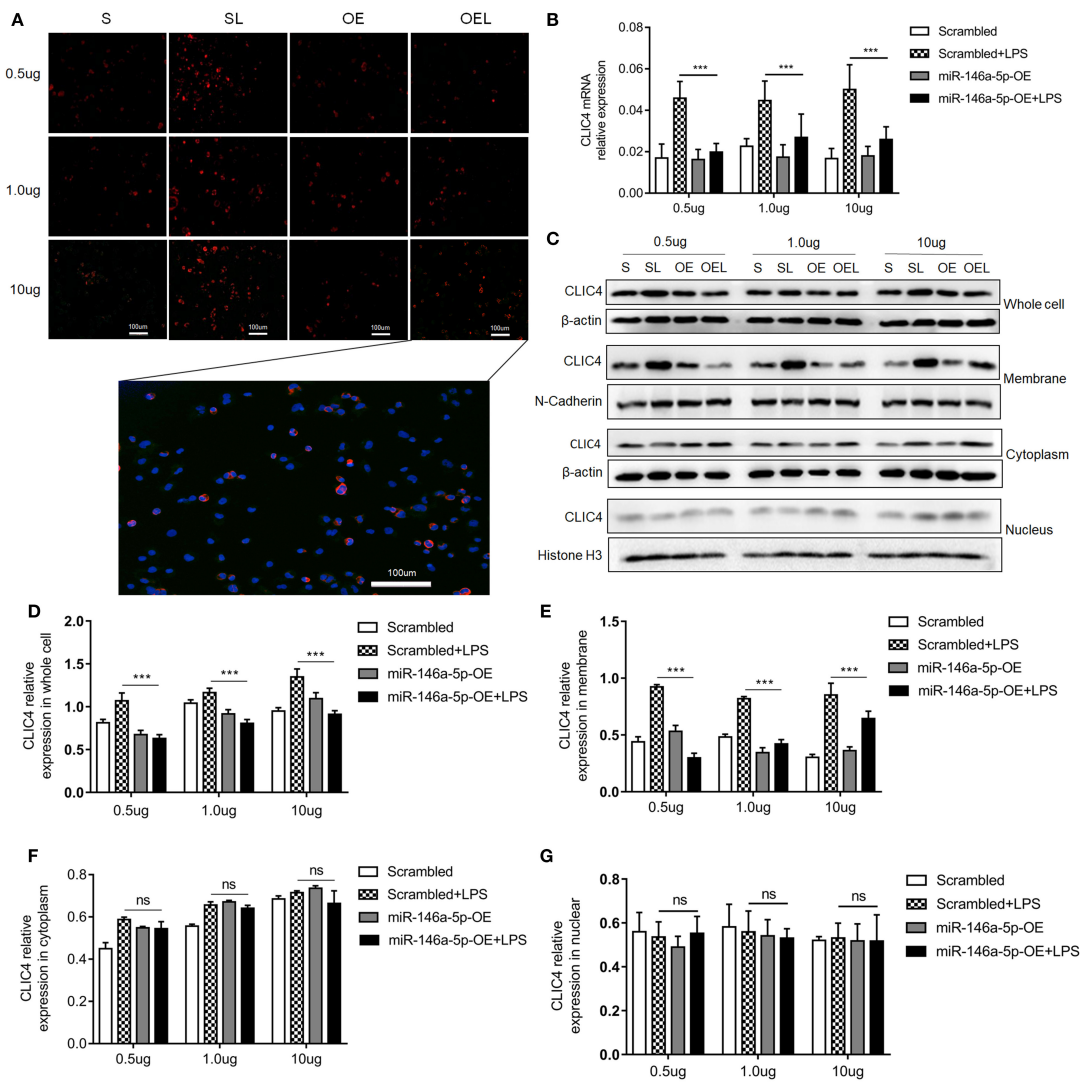
CLIC4 expression level in macrophage subcellular fraction after miR-146a-5p overexpression sequence pre-incubation followed by LPS/ATP stimulation. **(A)** Immunofluorescence showing the CLIC4 expression level after miR-146a-5p overexpression sequence pre-incubation followed by 0.5 μg/ml LPS + 5 mM ATP, 1.0 μg/ml LPS + 5 mM ATP, or 10 μg/ml LPS + 5 mM ATP stimulation. The representative merged picture of CLC4 and DAPI is shown at the bottom of panel A. **(B)** CLIC4 mRNA expression level after miR-146a-5p overexpression sequence pre-incubation followed by 0.5 μg/ml LPS + 5 mM ATP, 1.0 μg/ml LPS + 5 mM ATP, or 10 μg/ml LPS + 5 mM ATP stimulation (*n* = 6 per group). **(C)** CLIC4 protein expression levels in the whole cell, membrane, cytoplasm, and nucleus of macrophage after miR-146a-5p overexpression sequence pre-incubation followed by 0.5 μg/ml LPS + 5 mM ATP, 1.0 μg/ml LPS + 5 mM ATP, or 10 μg/ml LPS + 5 mM ATP stimulation. **(D–G)** The statistics of CLIC4 protein levels of **(C)** the whole cell, membrane, cytoplasm, and nucleus of macrophage (*n* = 4 per group). Scrambled (S), miR-146a-5p scrambled sequence; Scrambled +LPS (SL), miR-146a-5p scrambled sequence +LPS; miR-146a-5p-OE (OE), miR-146a-5p overexpression sequence; miR-146a-5p-OE+LPS (OEL), miR-146a-5p overexpression sequence +LPS. ***P < 0.001. ns, no statistical difference.

Numerous studies suggest that CLIC4 membrane expression is closely relative to LPS-induced NLRP3 activation (He et al., 2011; Malik et al., 2012; Domingo-Fernandez et al., 2017; Tang et al., 2017). The expression of CLIC4 was significantly increased in NEC human inflamed intestinal tissues, but not in NEC unaffected tissues and intestinal atresia tissues (Figure 5). Next, we investigated whether miR-146a-5p overexpression inhibited LPS/ATP-induced CLIC4 expression level in membrane. The expression level of miR-146a-5p was confirmed after miR-146a-5p overexpression sequence incubation (Supplementary Figure 1). The expression of CLIC4 was increased after LPS/ATP incubation (Figure 6), and miR-146a-5p overexpression inhibited CLIC4 mRNA and protein expression (Figures 6A–C). However, the inhibitory effect of miR-146a-5p overexpression on CLIC4 was not as significant across the whole cell (Figure 6C, first band of the whole cell). Malik et al. showed that CLIC4 nuclear translocation regulated macrophage deactivation, while nuclear-targeted CLIC4 overexpression downregulated IL-β (Malik et al., 2012). To investigate these possibilities, we detected CLIC4 protein in the membrane, cytoplasm, and nucleus of the treated cells. CLIC4 protein was increased and mainly expressed on the membrane of the treated cells (Figure 6C). In addition, CLIC4 was significantly downregulated by miR-146a-5p mimics in the membrane and showed a more obvious difference than that in the whole cell, cytoplasm, and nucleus (Figures 6D–G).

Next, we sought to confirm miR-146a-5p knockdown's effect on CLIC4 after LPS/ATP incubation. The expression level of miR-146a-5p was confirmed after miR-146a-5p knockdown sequence incubation (Supplementary Figure 3A). PCR showed that miR-146a-5p knockdown had no effect on CLIC4 mRNA and protein expression (Supplementary Figures 3B,C). Western blot showed that miR-146a-5p knockdown had no effect on CLIC4 protein expression across the whole cell, membrane, cytoplasm, and nucleus (Supplementary Figures 3D–H).

Collectively, miR-146a-5p overexpression decreased NLRP3 inflammasome upstream protein CLIC4 membrane expression.

### MiR-146a-5p Overexpression Alleviated NEC by Inhibiting NLRP3 Inflammasome Downstream Inflammatory Factors and CLIC4 *In vivo*

**Figure 7:**
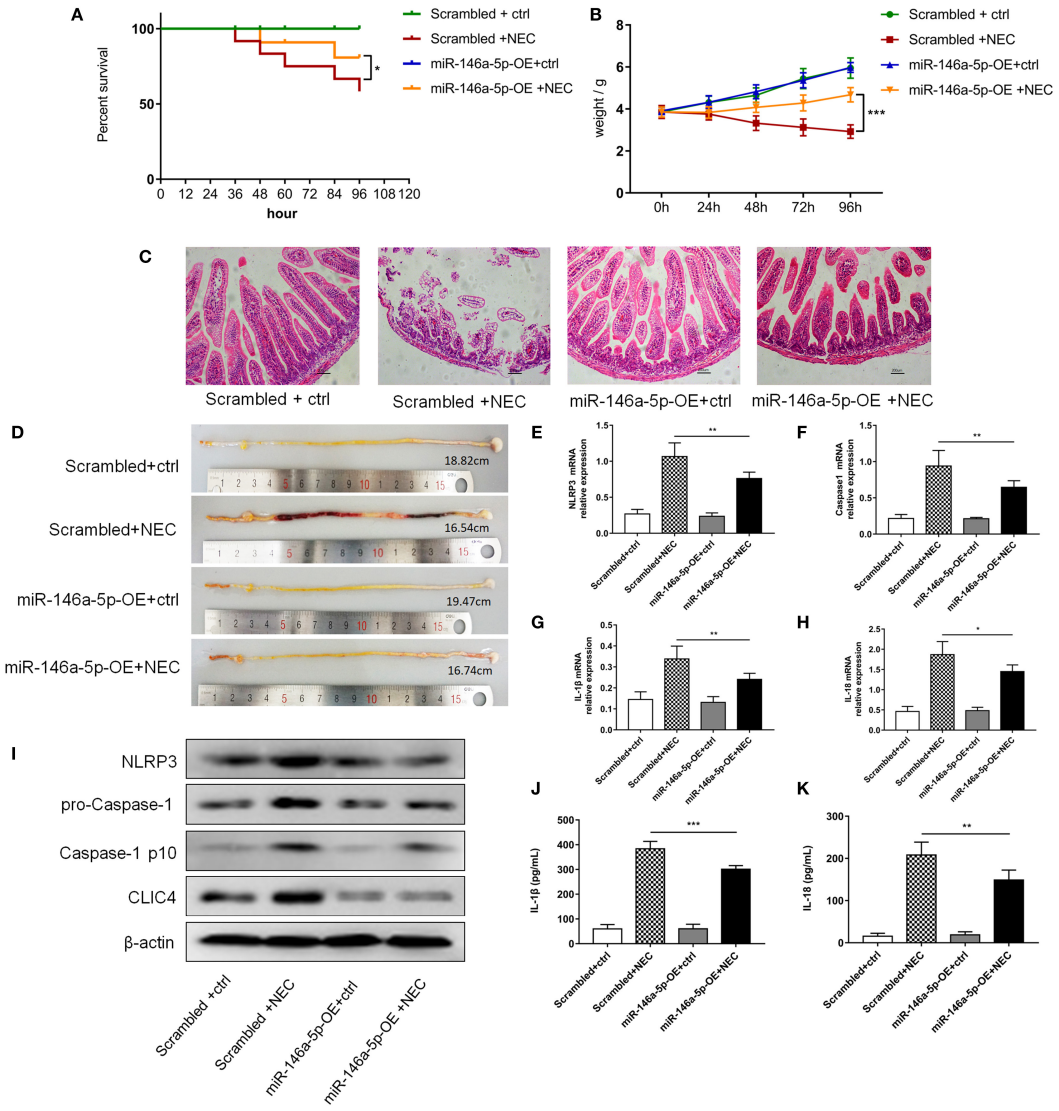
MiR-146a-5p overexpression adenovirus' effect on the activation of NLRP3 inflammasome and NEC mice. **(A)** Kaplan-Meier survival analysis of control and NEC mice after transduction with miR-146a-5p scrambled or overexpression adenovirus (*n* = 12 per group). **(B)** Body weight of control and NEC mice after miR-146a-5p scrambled or overexpression adenovirus transduction (*n* = 12 per group). **(C)** Representative pictures of HE in intestinal samples of control and NEC mice after transduction with miR-146a-5p scrambled or overexpression adenovirus. **(D)** Representative pictures of intestinal samples of control and NEC mice after miR-146a-5p scrambled or overexpression adenovirus transduction. **(E–H)** mRNA expression levels of NLRP3, Caspase-1, IL-1β, and IL-18 in intestinal samples of control and NEC mice after transduction with miR-146a-5p scrambled or overexpression adenovirus (*n* = 5 per group). **(I)** NLRP3, pro-Caspase-1, and Caspase-1 p10 protein expression levels in intestinal samples of control and NEC mice after transduction with miR-146a-5p scrambled or overexpression adenovirus. β-actin was re-used as the control image. **(J,K)** ELISA showing the IL-1β and IL-18 expression levels in intestinal samples of control and NEC mice after miR-146a-5p scrambled overexpression adenovirus transduction (*n* = 5 per group). Scrambled +ctrl, miR-146a-5p scrambled sequence adenovirus +control mice; Scrambled +NEC, miR-146a-5p scrambled sequence adenovirus +NEC mice; miR-146a-5p-OE +ctrl, miR-146a-5p overexpression adenovirus +control mice; miR-146a-5p-OE+NEC, miR-146a-5p overexpression adenovirus +NEC mice. *P < 0.05, **P < 0.01, ***P < 0.001.

To further validate the effects of miR-146a-5p in NEC development, we constructed a miR-146a-5p overexpression adenovirus. Neonatal mice were trans-ducted with the control and positive adenovirus by intraperitoneal injection. Then, these neonatal mice were randomly allocated to the control or NEC group. The miR-146a-5p expression level in the terminal ileum was confirmed (Supplementary Figure 4). The overall survival rate of the NEC group was significantly lower than that of the control group. MiR-146a-5p overexpression adenovirus transduction markedly improved the NEC survival rate (Figure 7A). In addition, miR-146a-5p attenuated the NEC-induced weight loss and alleviated intestinal damage (Figures 7B,C; Table 1). Representative images of gross morphology demonstrated that edema, congestion, necrosis, and reddish-black coloring were more severe in the Scrambled + NEC group than those in the miR-146a-5p-OE + NEC group (Figure 7D). These data indicated that miR-146a-5p protected neonatal mice from NEC intestinal injury and promoted digestive tract growth. Furthermore, miR-146a-5p significantly reduced NLRP3 inflammasome mRNA and protein (Figures 7E–I) expression as well as IL-1β, IL-18 (Figures 7G–K) secretion *in vivo*.

**Table 1:** Pathological grade of mouse terminal ileum.

**Group**	**Average grade score**	***n***	***P***
Scrambled +ctrl	0.17 ± 0.39	12	
Scrambled +NEC	3.25 ± 0.87	12	
miR-146a-5p-OE+ctrl	0.25 ± 0.45	12	
miR-146a-5p-OE+NEC	2.25 ± 1.22	12	^*^

*Average grade scores are shown as mean ± standard deviation. Scrambled +ctrl, miR-146a-5p scrambled adenovirus +control mice. Scrambled +NEC, miR-146a-5p scrambled adenovirus +NEC mice; miR-146a-5p-OE+ctrl, miR-146a-5p overexpression adenovirus +control mice; miR-146a-5p-OE+NEC, miR-146a-5p overexpression adenovirus NEC mice*.

* *(Scrambled +NEC vs. miR-146a-5p-OE+NEC) P < 0.05*.

In summary, all these results suggested a protective role of miR-146a-5p in NEC development by inhibiting NLRP3 inflammasome downstream inflammatory factors and CLIC4.

## Discussion

Our results identified the effect of miR-146a-5p/CLIC4/NLRP3 signaling in NEC development, which is a major cause of neonatal morbidity and mortality. First, we found that miR146a-5p was increased in the lamina propria of macrophages in NEC, and NLRP3 inflammasome activation was also increased in NEC inflammasome. Our data also demonstrated that macrophage NLRP3 inflammasome downstream inflammatory factors increased, and the inhibitory effects of miR-146a-5p on NLRP3 and CLIC4 were confirmed *in vivo*.

The leading pathogenic factors of NEC are various and unclear, including prematurity, low birth weight, bacterial colonization, hypoxia, and enteral feeding. Moreover, inflammation in NEC intestines is a complicated process, involving neutrophils, Treg cells, Th17 cells, and dendritic cells (Emami et al., 2011; Lam et al., 2013; Weitkamp et al., 2013). However, macrophages are thought to be the first cells that contribute to the maintenance of homeostasis, as well as the first to initiate an immune response during injury, as intestinal macrophages typically reside beneath the epithelial layer in the lamina propria (McElroy and Weitkamp, 2011; Mara et al., 2018). In addition, gut mucosal injury is marked by macrophage infiltration in contrast to pleomorphic infiltration in adults, especially in NEC (MohanKumar et al., 2012). Furthermore, macrophage concentration decrease in blood is identified as a marker for NEC in very low birth weight infants (Remon et al., 2014). In our study, macrophage infiltration in NEC intestinal lamina propria was confirmed.

Macrophages are considered to have several subtypes and have diverging responses to inflammasome activators. Although macrophages are categorized as inflammatory M1 macrophages and M2 macrophages and are associated with fibrosis and wound healing, this classification system may oversimplify the diversity of matured macrophages (Gordon, 2003; Mantovani et al., 2005; Pelegrin and Surprenant, 2009). Christian et al. found that NLRP3 and IL-1β both were increased in LPS priming M1 and M2 macrophages (Schmidt-Lauber et al., 2015). In contrast, Awad and colleagues demonstrated that the induction of NLRP3 expression by LPS is inhibited in the presence of IL-4+IL-13 (M2 phenotype) at both mRNA and protein levels in monocytes and macrophages, and that expression of Caspase-1 is upregulated in M1 cells, but not in M2 cells. However, the protein levels of pro-Caspase-1, pro-IL-1β, ASC, IL-1β, and IL-18 showed no significant differences in M1 and M2 macrophages (Awad et al., 2017). In our study, F4/80 was used as the macrophage marker rather than other subtype markers. *In vitro*, macrophages were incubated with LPS/ATP to simulate the macrophage-induced innate immune response in NEC intestines. Bauernfeind and colleagues reported that NLRP3 protein level was increased in a grade-distribution form of LPS concentration gradient (Bauernfeind et al., 2009). Consistent with these findings, our data demonstrated the increasing dependence of NLRP3, pro-Caspase-1, and Caspase-1 p10.

The protective effects of miR-146a in inflammation have been confirmed in numerous studies (Balasubramanyam et al., 2011; Song et al., 2017; Qu et al., 2019). Notably, miR-146a modulated NLRP3 inflammasome activation in the macrophage through TLR4-NF-κB signaling, in which TRAF6 and IRAK1 were definite target genes of miRA-146a (Chassin et al., 2012; Saba et al., 2014). In neonatal intestinal immune response, miR-146a mediates protective innate immune tolerance by targeting IRAK1 (Chassin et al., 2010). Immune tolerance is a status in which the immune system is in a state of non-reactivity to an antigen to prevent inflammatory tissue-destructive reactions (Knop and Knop, 2010). Our data revealed that miR-146a-5p was only able to inhibit the increasing of NLRP3 inflammasome enzymatic protein caspase-1 and downstream inflammatory factors under conditions with a relatively low and moderate concentration of LPS (0.5 μg/ml and 1.0 μg/ml). Additionally, miR-146a-5p mimic could not reduce the inflammatory factor to the control group level. Furthermore, miR-146a-5p overexpression adenovirus showed a protective role in NEC mice, yet it manifested a poor inhibitory effect on inflammatory factors compared with that shown by mimic showed *in vitro*. All the results revealed that miR-146a-5p-induced immune tolerance exerted protective effect to a limited extent. In other words, when inflammation is too severe and irreversible, miR-146a-induced immune tolerance will surpass the critical point and instead damage organs.

The classical mechanism of miRNA modulation occurs through binding to the 3′-UTR region of target gene. Thus, miRNA overexpression and knockdown should demonstrate an opposite regulating effect (O'Connell et al., 2012; Mohr and Mott, 2015). In our study, miR-146a-5p overexpression showed a significant inhibitory effect on NLRP3 inflammasome downstream inflammatory factors and CLIC4 membrane expression. However, NLRP3 inflammasome downstream inflammatory factors and CLIC4 membrane expression were not promoted by miR-146a knockdown. This may be a non-classical way for miR-146a to modulate target gene expression, which has been shown in other studies. Huang et al. found that miR-146a overexpression could suppress cell growth and increase cellular apoptosis in HCC cell lines, and miR-146a knockdown did not show a similar effect. In addition, only the role of miR-146a overexpression was investigated in several studies (Liu et al., 2017; Luo et al., 2019; Wang et al., 2019; Su et al., 2020). Considering the situation, miR-146a-5p knockdown adenovirus transduction was not conducted *in vivo*.

A remarkable feature of LPS/ATP-stimulated macrophages is cell-swelling (Perregaux et al., 1996), a response that is controlled by the coordinated action of K^+^ and Cl^−^ as well as the activity of multiple ion channels, a process known as regulatory volume decrease (Domingo-Fernandez et al., 2017; Tang et al., 2017). In previous studies, CLIC4 knock-out macrophages exhibit dysregulation of multiple inflammatory mediators during the early response to LPS (He et al., 2011; Malik et al., 2012), and CLIC4 nuclear-targeted overexpression may lead to decreased levels of IL-1β in stimulated macrophages (Malik et al., 2012). In contrast, mitochondrial reactive oxygen species then induce the translocation of CLICs to the plasma membrane for the induction of chloride efflux to promote NEK7–NLRP3 interaction (Tang et al., 2017). Domingo-Fernandez and colleagues revealed that upon LPS/ATP stimulation, CLIC1 and CLIC4 translocated into the cellular and nuclear membranes, and CLIC1 or CLIC4 knockdown impaired transcription of IL-1, ASC speck formation, and secretion of mature IL-1 (Domingo-Fernandez et al., 2017). Another study demonstrated that multiple stress inducers cause the translocation of cytoplasmic CLIC4 to the nucleus. Nevertheless, the direct influence of CLIC4 nuclear translocation on NLRP3 inflammasome had not been shown in this study (Suh et al., 2004). Our study is the first to find that miR-146a-5p regulates CLIC4 membrane expression. Despite these discoveries, there are some limitations to this study. The results will be more convincing if *in vitro* experiments are conducted in the primary macrophages of NEC mice, and the role of miR-146a-5p could be clarified more thoroughly using transgenic mice for conditional miR-146a-5p overexpression. In addition, the role of miR-146a-5p/NLRP3/CLIC4 in the epithelial cells of NEC patients merits further exploration.

In summary, we have identified miR-146a-5p as a protective factor in NEC development via inhibitingNLRP3 inflammasome downstream inflammatory factors. The interactions of miR-146a-5p, CLIC4, and NLRP3 inflammasome provide a new insight into the mechanisms of innate immune response, and the anti-inflammatory role of miR-146a-5p discovered by this study might indicate its potential as a therapeutic target in NEC.

## Data Availability Statement

The original contributions presented in the study are included in the article/Supplementary Material, further inquiries can be directed to the corresponding author.

## Ethics Statement

The studies involving human participants were reviewed and approved by Institutional Review Board at Shanghai Children's Hospital. Written informed consent to participate in this study was provided by the participants' legal guardian/next of kin. The animal study was reviewed and approved by Institutional Review Board of Shanghai Children' Hospital.

## Author Contributions

ZL and JC developed the experimental concept and design of the study. JC and XZ conducted the cell experiments. JC and TC conducted the animal model. JC and JZ conducted the FISH, IHC, and HE. JC and TC conducted the WB, Elisa, and PCR assays. JC, TC, and QS performed the statistical analyses. JC, QS, and TC drafted and revised the manuscript. ZL authorized the final version to be published. All authors have read and approved the final manuscript.

## Conflict of Interest

The authors declare that the research was conducted in the absence of any commercial or financial relationships that could be construed as a potential conflict of interest.

## Supplementary Material

Supplementary Figure 1 miR-146a-5p expression level in macrophages after miR-146a-5p overexpression sequence incubation (*n* = 6 per group). Scrambled, miR-146a-5p scrambled sequence; miR-146a-5p-OE, miR-146a-5p overexpression sequence. ^***^*P* < 0.001.

Supplementary Figure 2 The effects of miR-146a-5p knockdown sequence incubation on NLRP3 inflammasome activation level in macrophage after LPS/ATP stimulation. **(A–D)** mRNA expression levels of NLRP3, Caspase-1, IL1β, and IL-18 after miR-146a-5p knockdown sequence pre-incubation followed by 0.5 μg/ml LPS + 5 mM ATP or 1.0 μg/ml LPS + 5 mM ATP or 10 μg/ml LPS + 5 mM ATP stimulation (*n* = 6 per group). **(E–I)** ELISA showing IL-1β, IL-18, IL6, IL-10, and TNF-a expression levels after miR-146a-5p knockdown sequence pre-incubation followed by 0.5 μg/ml LPS + 5 mM ATP, 1.0 μg/ml LPS + 5 mM ATP, or 10 μg/ml LPS + 5 mM ATP stimulation (*n* = 6 per group). **(J)** NLRP3, pro-Caspase-1, and Caspase-1 p10 protein expression levels after miR-146a-5p knockdown sequence pre-incubation followed by 0.5 μg/ml LPS + 5 mM ATP, 1.0 μg/ml LPS + 5 mM ATP, or 10 μg/ml LPS + 5 mM ATP stimulation. β-actin was re-used as the control image. Scrambled (S), miR-146a-5p scrambled sequence; Scrambled +LPS (L), miR-146a-5p scrambled sequence +LPS; miR-146a-5p-KD(KD), miR-146a-5p knockdown sequence; miR-146a-5p-KD+LPS (KDL), miR-146a-5p knockdown sequence +LPS. ^*^*P* < 0.05, ^***^*P* < 0.001. ns, no statistical difference.

Supplementary Figure 3 CLIC4 expression level in macrophage subcellular fraction after miR-146a-5p knockdown sequence pre-incubation followed by LPS/ATP stimulation. **(A)** miR-146a-5p expression level in macrophages after miR-146a-5p knockdown sequence pre-incubation (*n* = 6 per group). **(B)** CLIC4 mRNA expression level after inhibitor knockdown sequence pre-incubation followed by 0.5 μg/ml LPS + 5 mM ATP, 1.0 μg/ml LPS + 5 mM ATP, or 10 μg/ml LPS + 5 mM ATP stimulation (*n* = 6 per group). **(C)** Immunofluorescence showing the CLIC4 expression level after miR-146a-5p knockdown sequence pre-incubation followed by 0.5 μg/ml LPS + 5 mM ATP or 1.0 μg/ml LPS + 5 mM ATP or 10 μg/ml LPS + 5 mM ATP stimulation. **(D)** CLIC4 protein expression levels in the whole cell, membrane, cytoplasm, and nucleus of macrophage after miR-146a-5p knockdown sequence pre-incubation followed by 0.5 μg/ml LPS + 5 mM ATP or 1.0 μg/ml LPS + 5 mM ATP or 10 μg/ml LPS + 5 mM ATP stimulation. **(E–H)** The statistics of CLIC4 protein levels of **(D)** in the whole cell, membrane, cytoplasm, and nucleus of macrophage (*n* = 4 per group). Scrambled (S), miR-146a-5p scrambled sequence; Scrambled +LPS (SL), miR-146a-5p scrambled sequence +LPS; miR-146a-5p-KD (KD), miR-146a-5p knockdown sequence; miR-146a-5p-KD +LPS (KDL), miR-146a-5p knockdown sequence +LPS. ^***^*P* < 0.001. ns, no statistical difference.

Supplementary Figure 4 miR-146a-5p mRNA expression in the terminal ileum tissue from mice with miR-146a-5p overexpression adenovirus transduction (*n* = 5 per group). Scrambled +ctrl, miR-146a-5p scrambled sequence adenovirus +control mice; Scrambled +NEC, miR-146a-5p scrambled sequence adenovirus +NEC mice; miR-146a-5p-OE +ctrl, miR-146a-5p overexpression adenovirus +control mice; miR-146a-5p-OE+NEC, miR-146a-5p overexpression adenovirus +NEC mice. ^***^*P* < 0.001.
