# Supplementary figures and images for: MiR-146a-5p Mimic Inhibits NLRP3 Inflammasome Downstream Inflammatory Factors and CLIC4 in Neonatal Necrotizing Enterocolitis
Source: Front Cell Dev Biol. 2021 Jan 28;8:594143. doi: 10.3389/fcell.2020.594143 (PMC7876392; doi:10.3389/fcell.2020.594143)

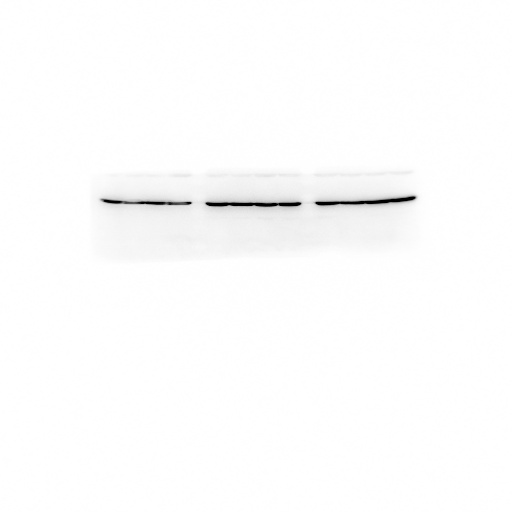

Supplement: Supplementary file 1 [file Data_Sheet_1.ZIP › ORIGINAL GEL IMAGES/b-actin-cytoplasm-inhibitor.jpg]

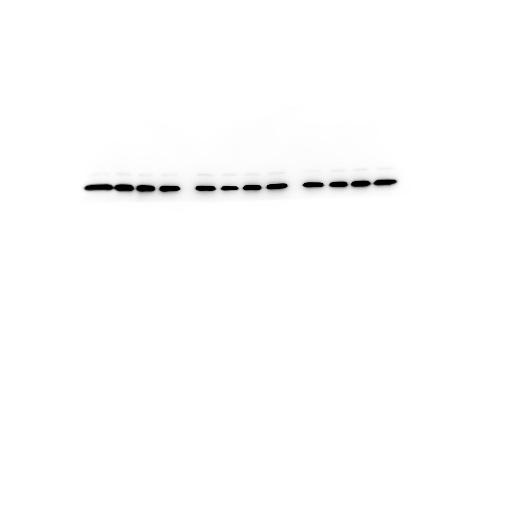

Supplement: Supplementary file 1 [file Data_Sheet_1.ZIP › ORIGINAL GEL IMAGES/b-actin-cytoplasm-mimc.jpg]

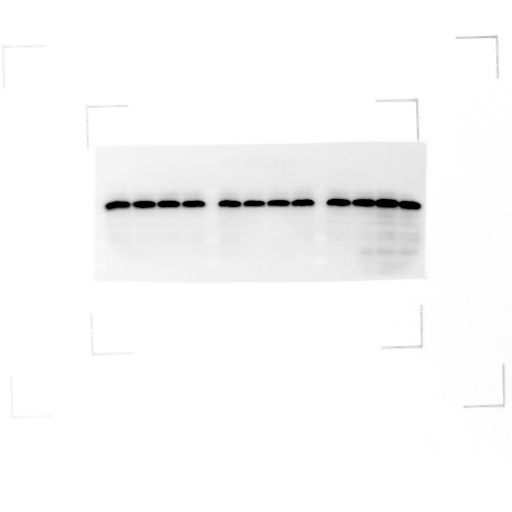

Supplement: Supplementary file 1 [file Data_Sheet_1.ZIP › ORIGINAL GEL IMAGES/b-actin-inhibitor.jpg]

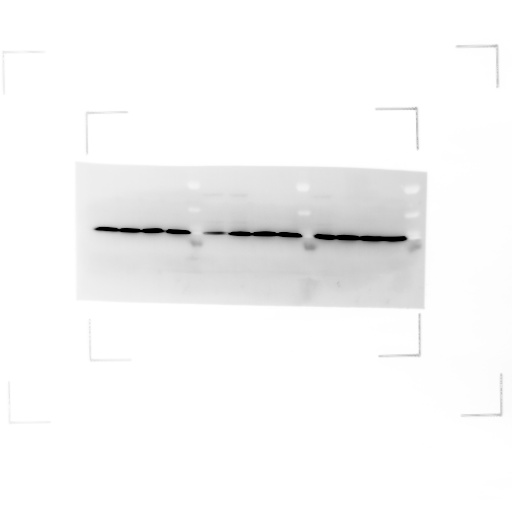

Supplement: Supplementary file 1 [file Data_Sheet_1.ZIP › ORIGINAL GEL IMAGES/b-actin-mimic+nec.jpg]

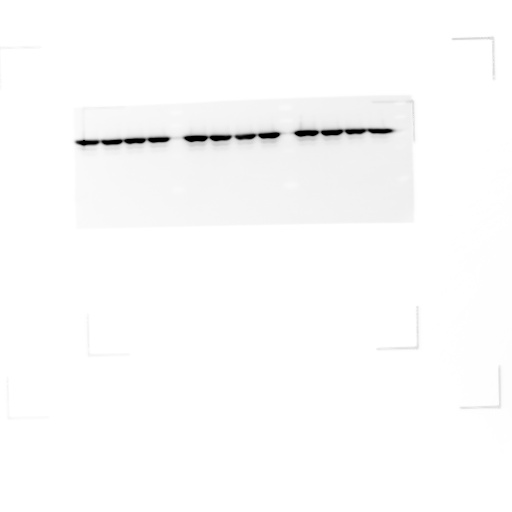

Supplement: Supplementary file 1 [file Data_Sheet_1.ZIP › ORIGINAL GEL IMAGES/b-actin-mimic.jpg]

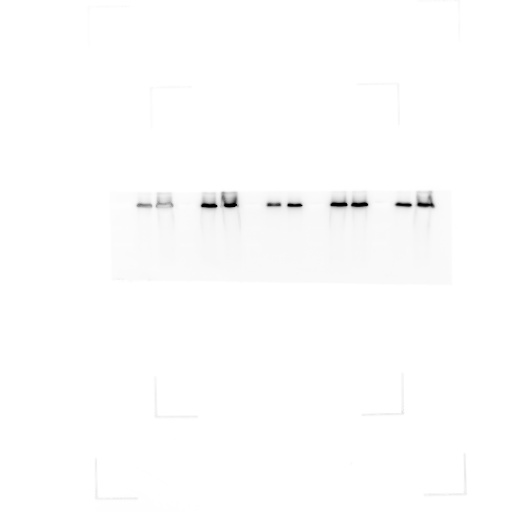

Supplement: Supplementary file 1 [file Data_Sheet_1.ZIP › ORIGINAL GEL IMAGES/b-ACTIN-NEC and control.jpg]

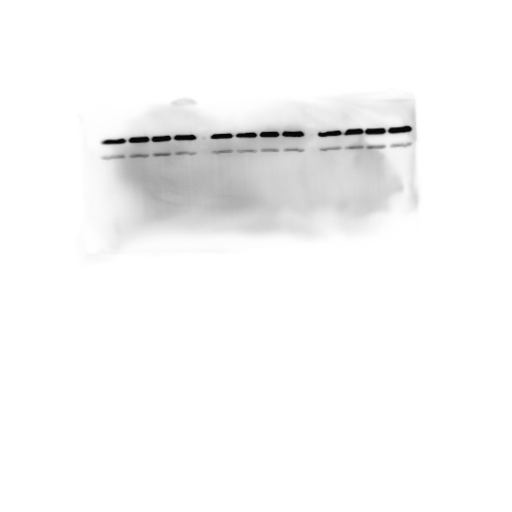

Supplement: Supplementary file 1 [file Data_Sheet_1.ZIP › ORIGINAL GEL IMAGES/b-actin-whole cell -mimic.jpg]

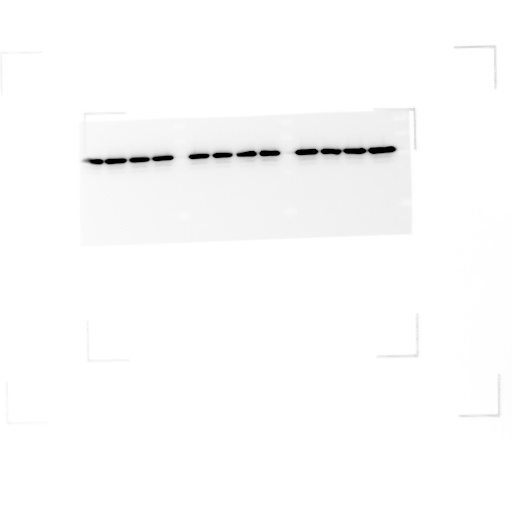

Supplement: Supplementary file 1 [file Data_Sheet_1.ZIP › ORIGINAL GEL IMAGES/b-actin-whole cell-inhibitor.jpg]

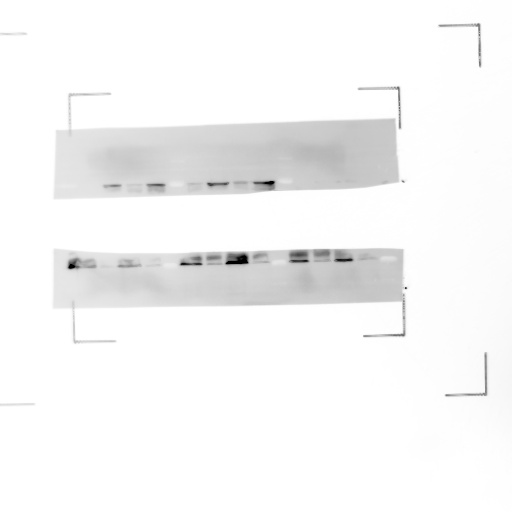

Supplement: Supplementary file 1 [file Data_Sheet_1.ZIP › ORIGINAL GEL IMAGES/casp1-p10 of NEC and control mouse.jpg]

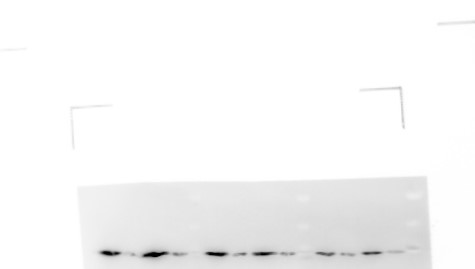

Supplement: Supplementary file 1 [file Data_Sheet_1.ZIP › ORIGINAL GEL IMAGES/CASP1-p10-inhibitor.jpg]

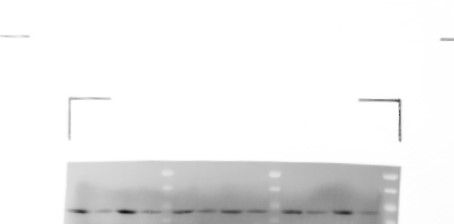

Supplement: Supplementary file 1 [file Data_Sheet_1.ZIP › ORIGINAL GEL IMAGES/casp1-p10-mimic+nec.jpg]

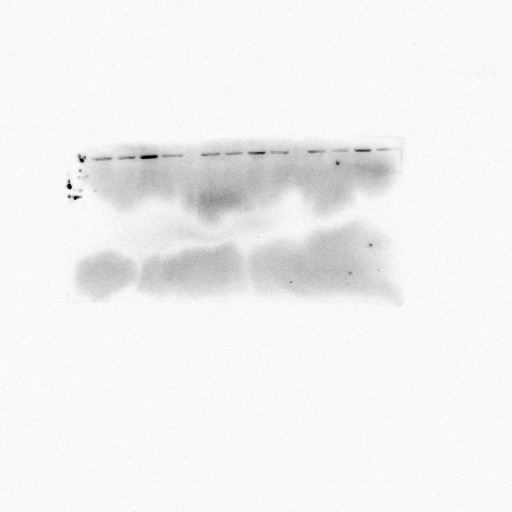

Supplement: Supplementary file 1 [file Data_Sheet_1.ZIP › ORIGINAL GEL IMAGES/casp1-p10-mimic.jpg]

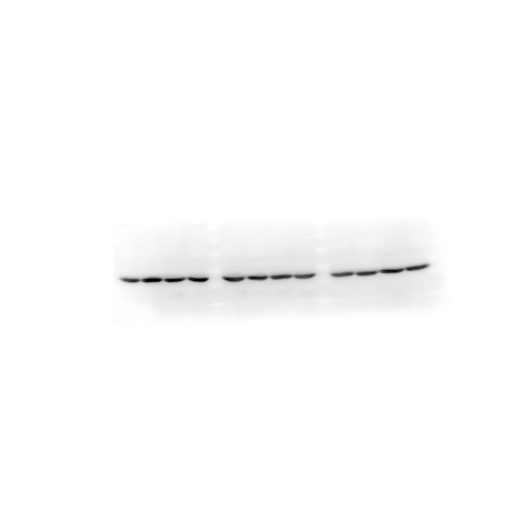

Supplement: Supplementary file 1 [file Data_Sheet_1.ZIP › ORIGINAL GEL IMAGES/CLIC4-cytoplasm-inhibitor.jpg]

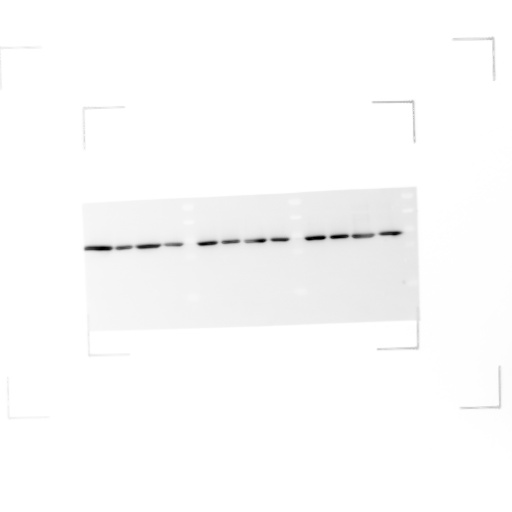

Supplement: Supplementary file 1 [file Data_Sheet_1.ZIP › ORIGINAL GEL IMAGES/CLIC4-cytoplasm-mimic.jpg]

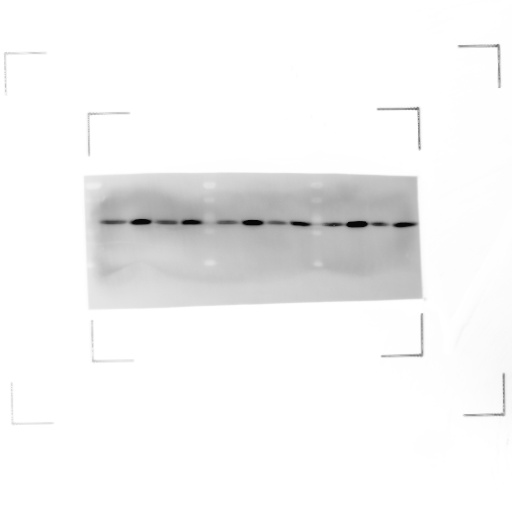

Supplement: Supplementary file 1 [file Data_Sheet_1.ZIP › ORIGINAL GEL IMAGES/CLIC4-membrane-inhibitor.jpg]

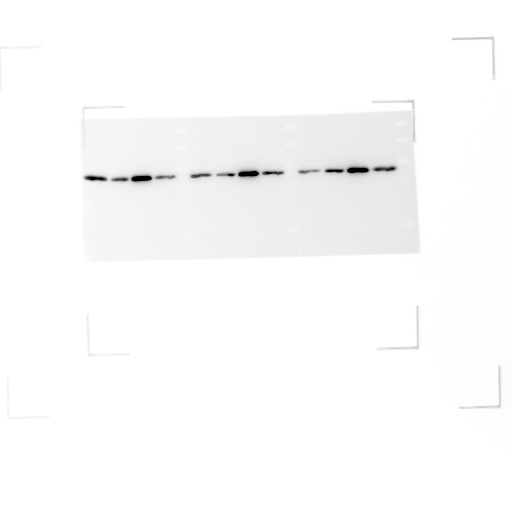

Supplement: Supplementary file 1 [file Data_Sheet_1.ZIP › ORIGINAL GEL IMAGES/CLIC4-membrane-mimic.jpg]

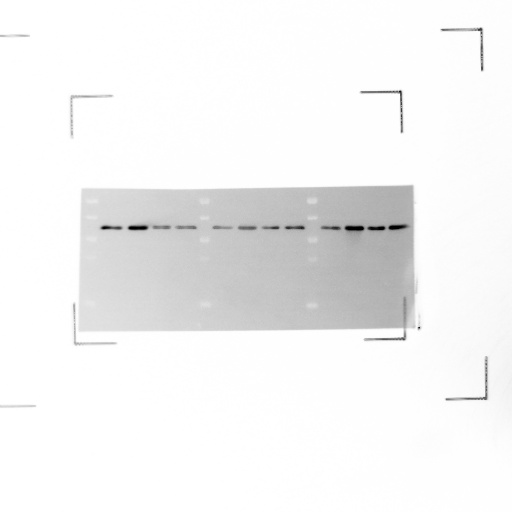

Supplement: Supplementary file 1 [file Data_Sheet_1.ZIP › ORIGINAL GEL IMAGES/CLIC4-mimic+NEC.jpg]

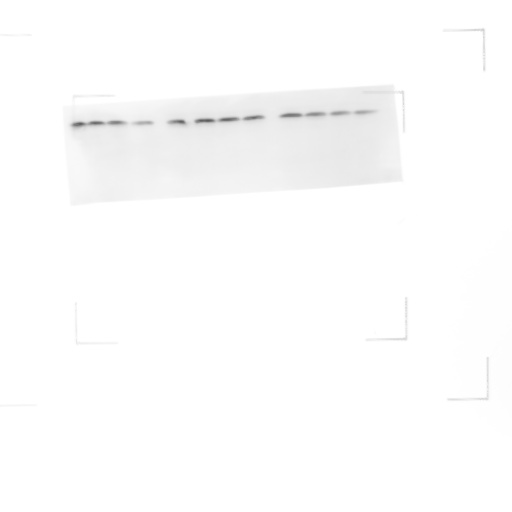

Supplement: Supplementary file 1 [file Data_Sheet_1.ZIP › ORIGINAL GEL IMAGES/CLIC4-nucleus-inhibitor.jpg]

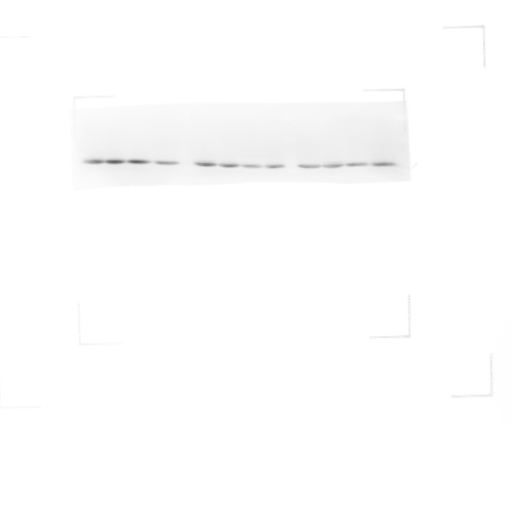

Supplement: Supplementary file 1 [file Data_Sheet_1.ZIP › ORIGINAL GEL IMAGES/CLIC4-nucleus-mimic.jpg]

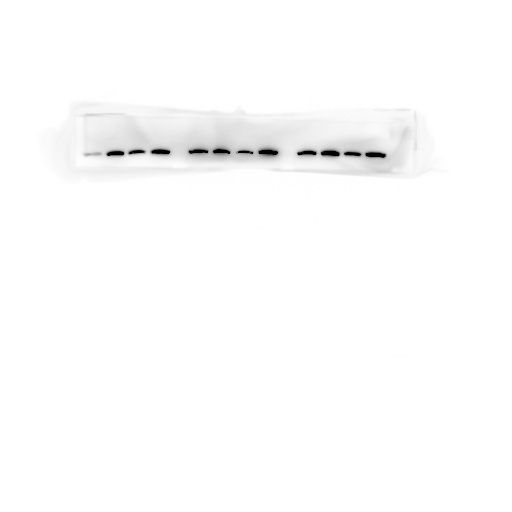

Supplement: Supplementary file 1 [file Data_Sheet_1.ZIP › ORIGINAL GEL IMAGES/CLIC4-whole cell-inhibitor.jpg]

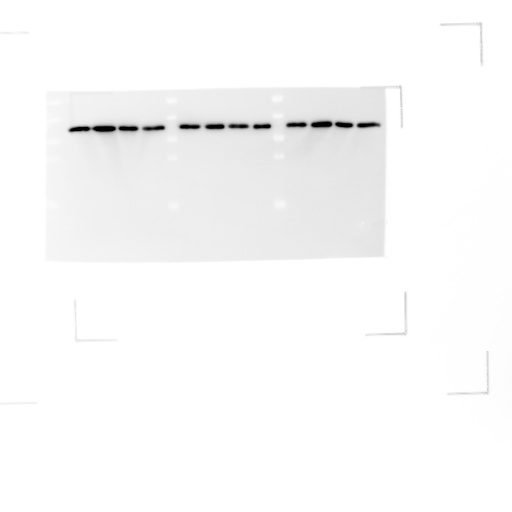

Supplement: Supplementary file 1 [file Data_Sheet_1.ZIP › ORIGINAL GEL IMAGES/CLIC4-whole-mimic.jpg]

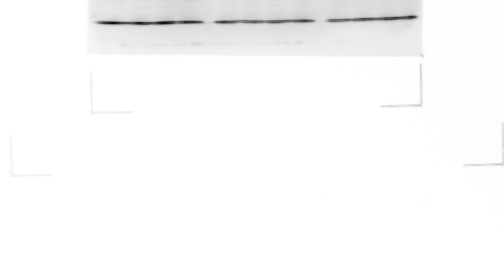

Supplement: Supplementary file 1 [file Data_Sheet_1.ZIP › ORIGINAL GEL IMAGES/H3-Inhibitor.jpg]

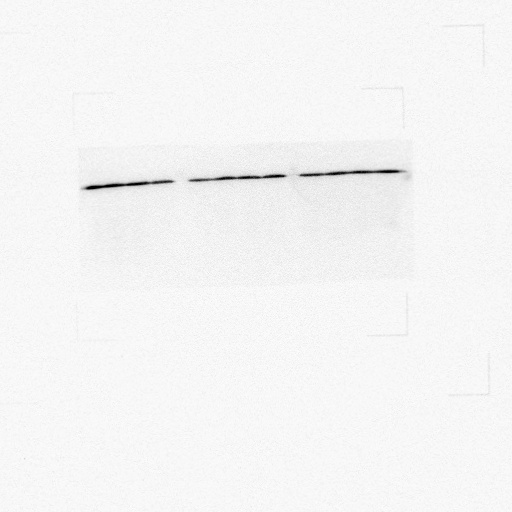

Supplement: Supplementary file 1 [file Data_Sheet_1.ZIP › ORIGINAL GEL IMAGES/H3-mimic.jpg]

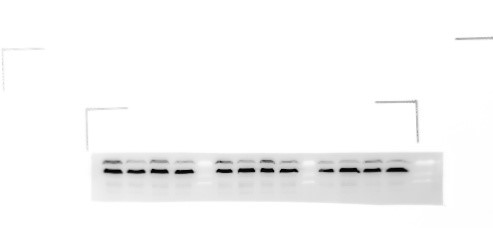

Supplement: Supplementary file 1 [file Data_Sheet_1.ZIP › ORIGINAL GEL IMAGES/NCAD-inhibitor.jpg]

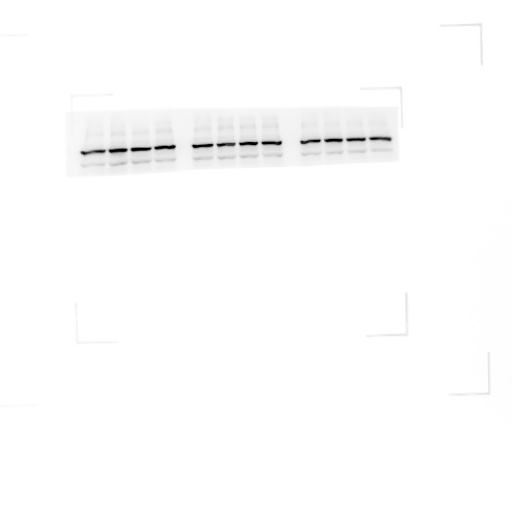

Supplement: Supplementary file 1 [file Data_Sheet_1.ZIP › ORIGINAL GEL IMAGES/NCAD-mimic.jpg]

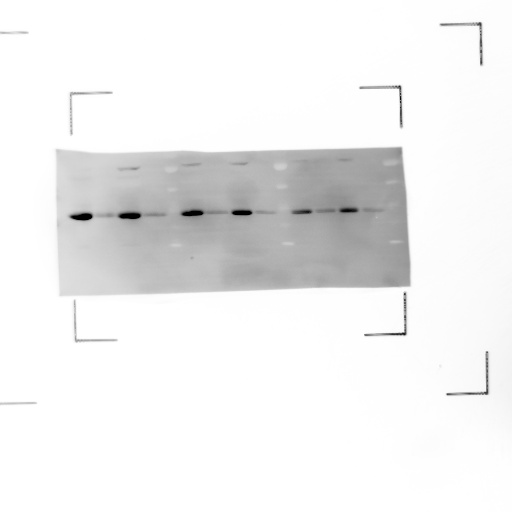

Supplement: Supplementary file 1 [file Data_Sheet_1.ZIP › ORIGINAL GEL IMAGES/NLRP3-inhibitor.jpg]

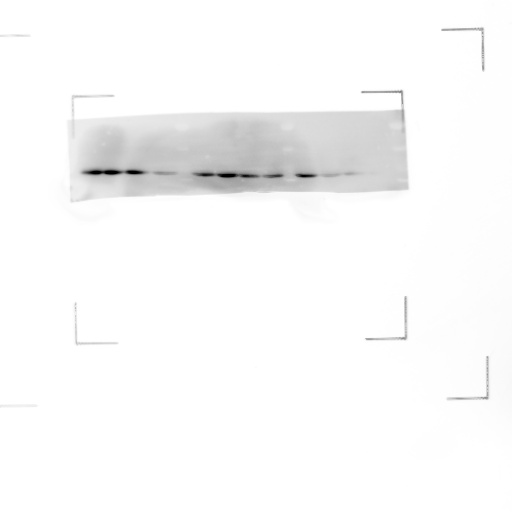

Supplement: Supplementary file 1 [file Data_Sheet_1.ZIP › ORIGINAL GEL IMAGES/NLRP3-mimic+nec.jpg]

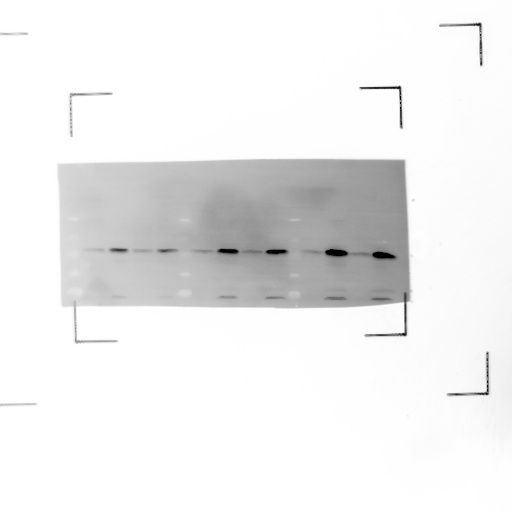

Supplement: Supplementary file 1 [file Data_Sheet_1.ZIP › ORIGINAL GEL IMAGES/NLRP3-mimic.jpg]

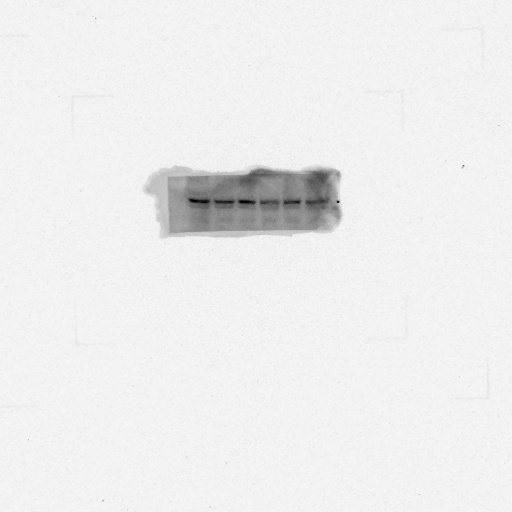

Supplement: Supplementary file 1 [file Data_Sheet_1.ZIP › ORIGINAL GEL IMAGES/NLRP3-NEC mouse and control.jpg]

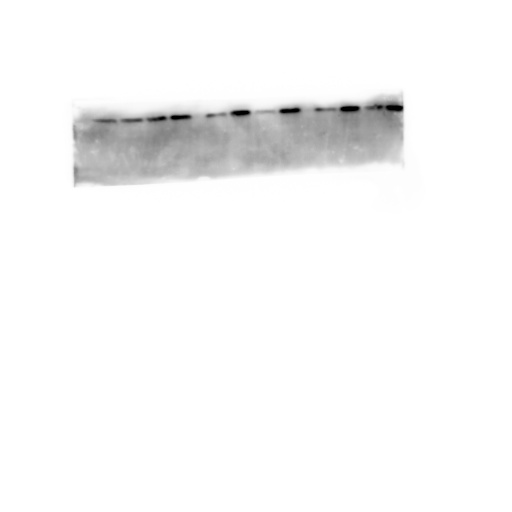

Supplement: Supplementary file 1 [file Data_Sheet_1.ZIP › ORIGINAL GEL IMAGES/pro-casp1-inhibitor.jpg]

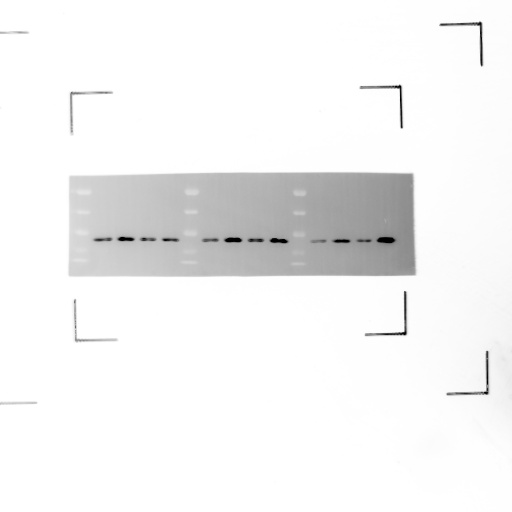

Supplement: Supplementary file 1 [file Data_Sheet_1.ZIP › ORIGINAL GEL IMAGES/pro-casp1-mimic+nec.jpg]

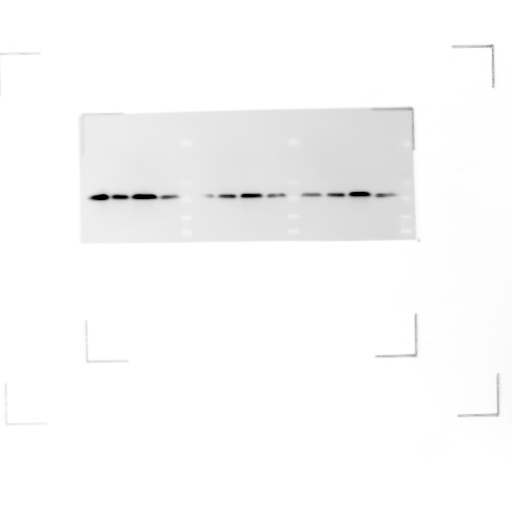

Supplement: Supplementary file 1 [file Data_Sheet_1.ZIP › ORIGINAL GEL IMAGES/pro-casp1-mimic.jpg]

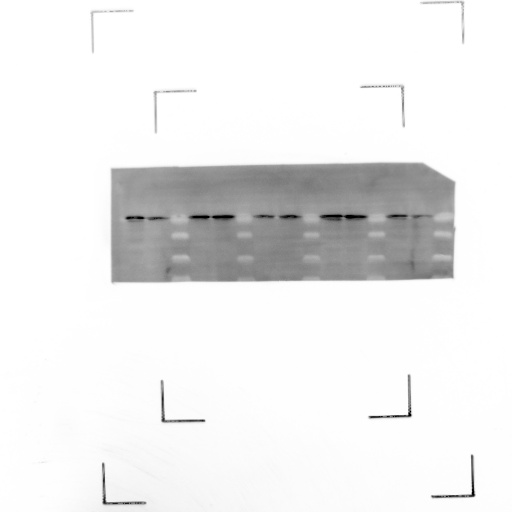

Supplement: Supplementary file 1 [file Data_Sheet_1.ZIP › ORIGINAL GEL IMAGES/pro-CASP1-NEC and control mouse.jpg]

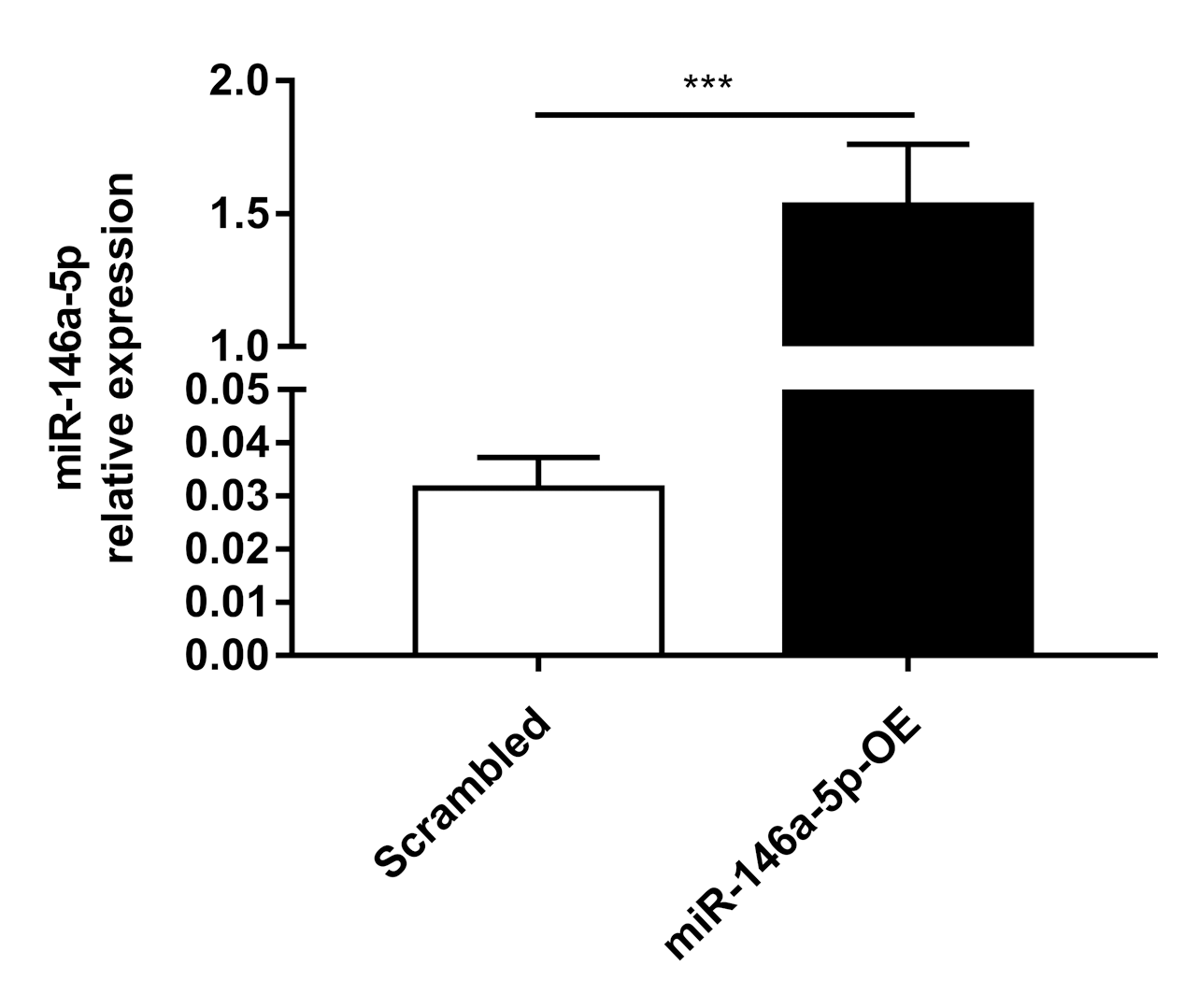

Supplement: Supplementary Figure 1 — miR-146a-5p expression level in macrophages after miR-146a-5p overexpression sequence incubation (n = 6 per group). Scrambled, miR-146a-5p scrambled sequence; miR-146a-5p-OE, miR-146a-5p overexpression sequence. ***P < 0.001. [file Image_1.TIF]

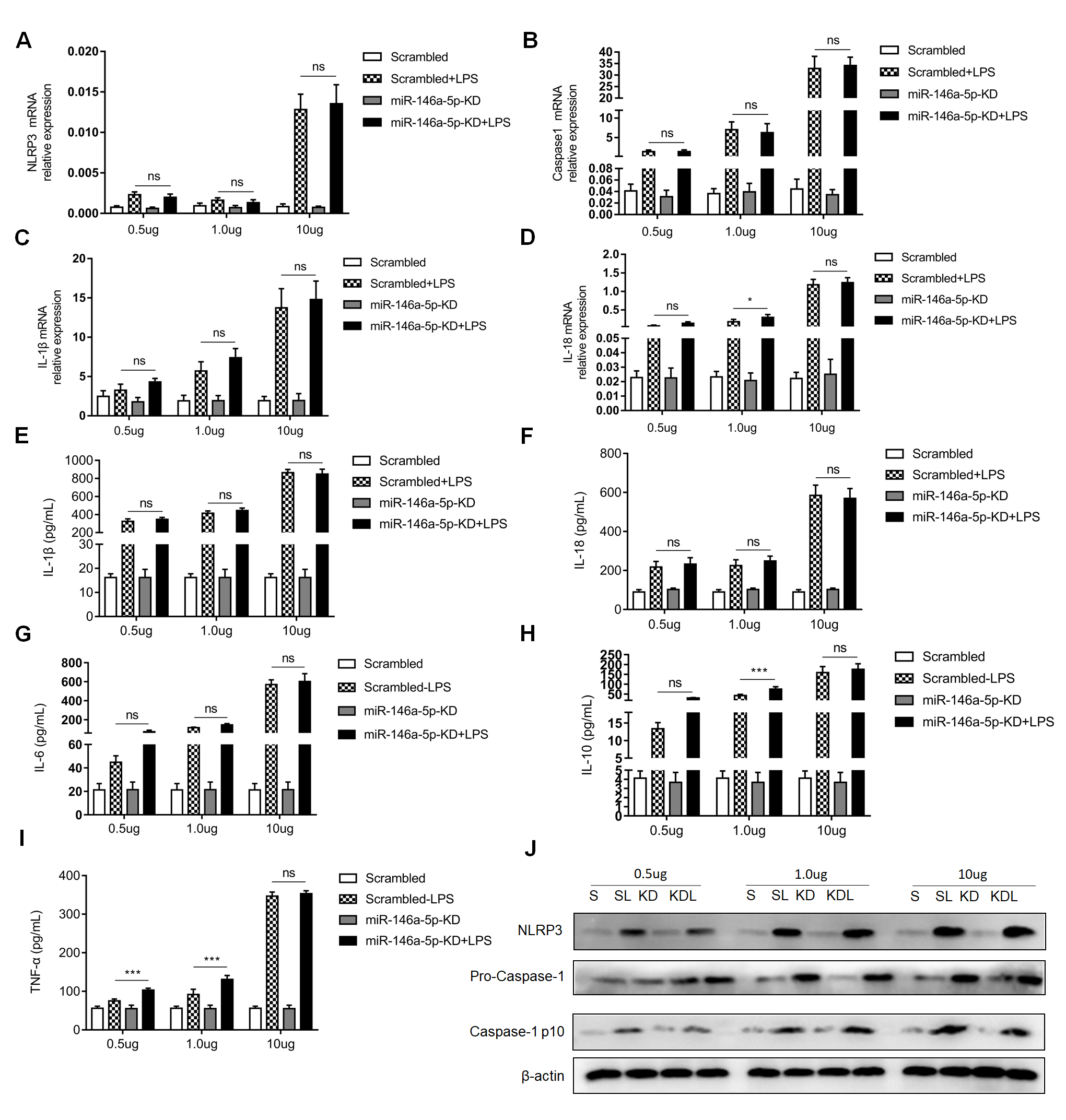

Supplement: Supplementary Figure 2 — The effects of miR-146a-5p knockdown sequence incubation on NLRP3 inflammasome activation level in macrophage after LPS/ATP stimulation. (A–D) mRNA expression levels of NLRP3, Caspase-1, IL1β, and IL-18 after miR-146a-5p knockdown sequence pre-incubation followed by 0.5 μg/ml LPS + 5 mM ATP or 1.0 μg/ml LPS + 5 mM ATP or 10 μg/ml LPS + 5 mM ATP stimulation (n = 6 per group). (E–I) ELISA showing IL-1β, IL-18, IL6, IL-10, and TNF-a expression levels after miR-146a-5p knockdown sequence pre-incubation followed by 0.5 μg/ml LPS + 5 mM ATP, 1.0 μg/ml LPS + 5 mM ATP, or 10 μg/ml LPS + 5 mM ATP stimulation (n = 6 per group). (J) NLRP3, pro-Caspase-1, and Caspase-1 p10 protein expression levels after miR-146a-5p knockdown sequence pre-incubation followed by 0.5 μg/ml LPS + 5 mM ATP, 1.0 μg/ml LPS + 5 mM ATP, or 10 μg/ml LPS + 5 mM ATP stimulation. β-actin was re-used as the control image. Scrambled (S), miR-146a-5p scrambled sequence; Scrambled +LPS (L), miR-146a-5p scrambled sequence +LPS; miR-146a-5p-KD(KD), miR-146a-5p knockdown sequence; miR-146a-5p-KD+LPS (KDL), miR-146a-5p knockdown sequence +LPS. *P < 0.05, ***P < 0.001. ns, no statistical difference. [file Image_2.TIF]

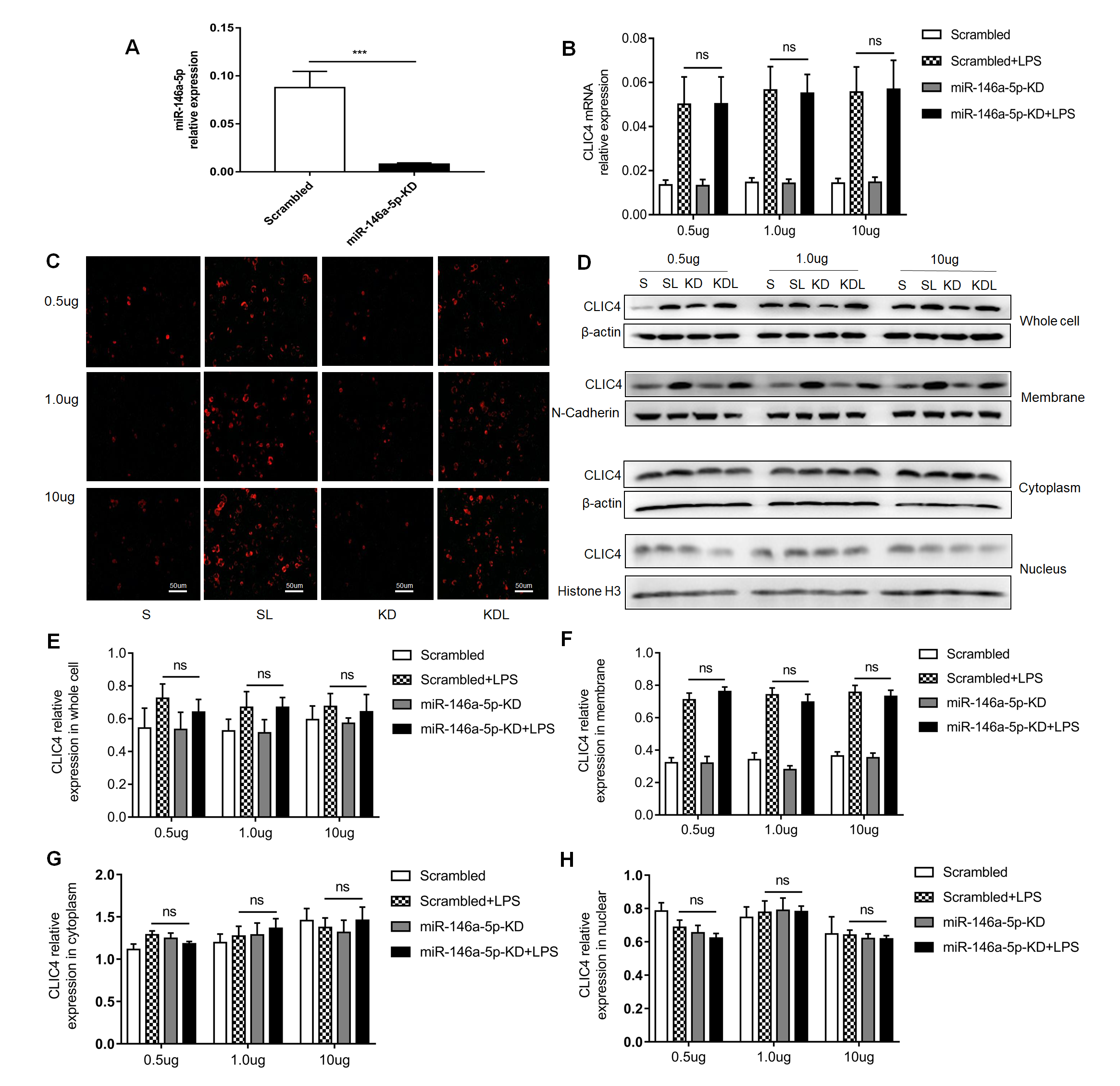

Supplement: Supplementary Figure 3 — CLIC4 expression level in macrophage subcellular fraction after miR-146a-5p knockdown sequence pre-incubation followed by LPS/ATP stimulation. (A) miR-146a-5p expression level in macrophages after miR-146a-5p knockdown sequence pre-incubation (n = 6 per group). (B) CLIC4 mRNA expression level after inhibitor knockdown sequence pre-incubation followed by 0.5 μg/ml LPS + 5 mM ATP, 1.0 μg/ml LPS + 5 mM ATP, or 10 μg/ml LPS + 5 mM ATP stimulation (n = 6 per group). (C) Immunofluorescence showing the CLIC4 expression level after miR-146a-5p knockdown sequence pre-incubation followed by 0.5 μg/ml LPS + 5 mM ATP or 1.0 μg/ml LPS + 5 mM ATP or 10 μg/ml LPS + 5 mM ATP stimulation. (D) CLIC4 protein expression levels in the whole cell, membrane, cytoplasm, and nucleus of macrophage after miR-146a-5p knockdown sequence pre-incubation followed by 0.5 μg/ml LPS + 5 mM ATP or 1.0 μg/ml LPS + 5 mM ATP or 10 μg/ml LPS + 5 mM ATP stimulation. (E–H) The statistics of CLIC4 protein levels of (D) in the whole cell, membrane, cytoplasm, and nucleus of macrophage (n = 4 per group). Scrambled (S), miR-146a-5p scrambled sequence; Scrambled +LPS (SL), miR-146a-5p scrambled sequence +LPS; miR-146a-5p-KD (KD), miR-146a-5p knockdown sequence; miR-146a-5p-KD +LPS (KDL), miR-146a-5p knockdown sequence +LPS. ***P < 0.001. ns, no statistical difference. [file Image_3.TIF]

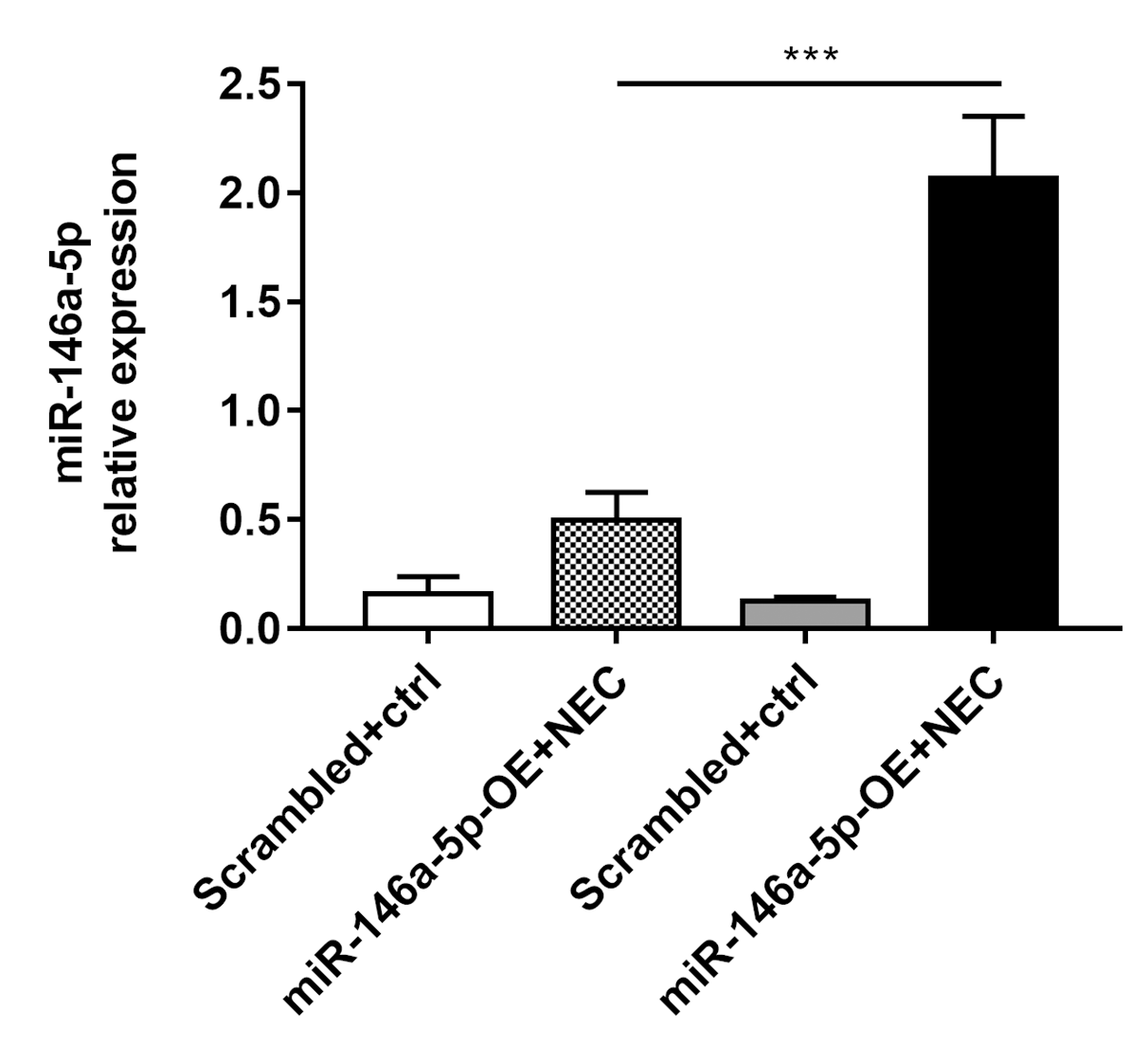

Supplement: Supplementary Figure 4 — miR-146a-5p mRNA expression in the terminal ileum tissue from mice with miR-146a-5p overexpression adenovirus transduction (n = 5 per group). Scrambled +ctrl, miR-146a-5p scrambled sequence adenovirus +control mice; Scrambled +NEC, miR-146a-5p scrambled sequence adenovirus +NEC mice; miR-146a-5p-OE +ctrl, miR-146a-5p overexpression adenovirus +control mice; miR-146a-5p-OE+NEC, miR-146a-5p overexpression adenovirus +NEC mice. ***P < 0.001. [file Image_4.TIF]
